# A dataset of asymptomatic human gait and movements obtained from markers, IMUs, insoles and force plates

**DOI:** 10.1038/s41597-023-02077-3

**Published:** 2023-03-30

**Authors:** Gautier Grouvel, Lena Carcreff, Florent Moissenet, Stéphane Armand

**Affiliations:** 1 Kinesiology Laboratory, Geneva University Hospitals and University of Geneva, Geneva, Switzerland; 2 Biomechanics Laboratory, Geneva University Hospitals and University of Geneva, Geneva, Switzerland

**Keywords:** Outcomes research, Quality of life, Databases

## Abstract

Human motion capture and analysis could be made easier through the use of wearable devices such as inertial sensors and/or pressure insoles. However, many steps are still needed to reach the performance of optoelectronic systems to compute kinematic parameters. The proposed dataset has been established on 10 asymptomatic adults. Participants were asked to walk at different speeds on a 10-meters walkway in a laboratory and to perform different movements such as squats or knee flexion/extension tasks. Three-dimensional trajectories of 69 reflective markers placed according to a conventional full body markerset, acceleration and angular velocity signals of 8 inertial sensors, pressure signals of 2 insoles, 3D ground reaction forces and moments obtained from 3 force plates were simultaneously recorded. Eight calculated virtual markers related to joint centers were also added to the dataset. This dataset contains a total of 337 trials including static and dynamic tasks for each participant. Its purpose is to enable comparisons between various motion capture systems and stimulate the development of new methods for gait analysis.

## Background & Summary

Human motion measurement and analysis represent a major topic in the medical field to understand people’s gross motor function, e.g. to identify possible risks of fall^1^ or to understand gait deviations^2^. This is usually performed with an optoelectronic system in a gait laboratory. However, this method requires qualified staff (especially for the marker placement which is done by palpation by an examiner and which is the largest source of variability^3^), advanced and expensive technological equipments^4^, and is time-consuming. These factors usually restrain such analyses to large medical institutions. In recent years, wearable systems have been trusted into the forefront of movement analysis due to their miniaturization. This is particularly the case for inertial sensors, also called Inertial Measurement Units (IMUs), that are commonly composed of a 3-axis accelerometer, a 3-axis gyroscope and eventually a 3-axis magnetometer^5^. By attaching these sensors to a set of body segments, they could give access to a quick, easy-to-use and less expensive gait analysis than an optoelectronic system^4^. Their use is increasingly popular in human movement science as evidenced by many studies in the field^6^.

Few datasets have been made available in the literature and can be used to validate the different steps of 3D kinematic calculations^7,8^. The measurements carried out in the present study provide a comprehensive dataset^9^ on asymptomatic participants with the 3D trajectories of 37 cutaneous markers, 32 cluster markers and 8 calculated joint centers, the signals of 8 IMUs placed on each lower-limb segment, on the pelvis and on the torso, the signals of 2 insoles, and the signals of 3 force plates installed in the middle of the walkway. The two last devices give also access to kinetic data and open comparison with other studies^10^. Various movements have been recorded, such as gait at different speeds (i.e. slow gait, comfortable gait, fast gait), running, squats and functional tasks.

The primary objective of this study was to provide a dataset^9^ allowing the comparison, validation, and improvement of different wearable motion capture systems and could lead to the development of a clinical protocol for clinical gait analysis outside the laboratory^11^. In particular, the resulting dataset is oriented towards the computation of lower-limb kinematics using IMUs, the computation of spatio-temporal parameters using instrumented insoles, and the development of activity detection algorithms using IMUs and insoles data, useful in many applications^12–15^. This work was carried out during the study of Carcreff *et al*.^16^. With the knowledge of the position and orientation of the IMU sensors in the optoelectronic camera coordinate system, the dataset was used to validate algorithms for calculating kinematics from inertial data^16^. This dataset^9^ could thus be used alone, or merged with another dataset, to validate such procedures and methods.

## Methods

### Participants

Ten asymptomatic participants (4 women and 6 men, 29.7 ± 6.4 years, 1.74 ± 0.06 m, 68.0 ± 13.9 kg) were recruited on a voluntary basis. The study was approved by the Ordinance on Human Research^17^ (project ID: CCER-2020-00358) with the exception of Clinical trials and follows the Swiss legal requirements, the current version of the World Medical Association Declaration of Helsinki and the principles of Good Clinical Practice. All participants gave their informed consent prior to their participation in the study. They were included if they were between 5 and 70 years old, asymptomatic (i.e. healthy without any disease affecting gait), with no previous surgery on lower limbs nor spine in the last two years, no allergy to hypoallergenic adhesive tape, and no known pregnancy.

#### Records

**Fig. 1:**
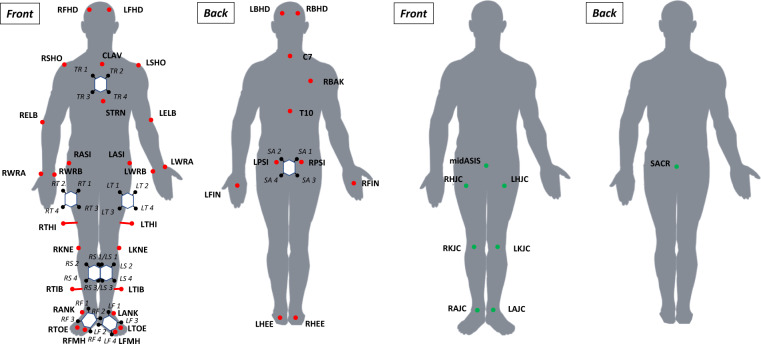
Reflective markers of the lower body placed according to the Conventional Gait Model 1.0 + reflective markers of the upper body placed according to the Plug In Gait model + LFMH marker (red markers), cluster markers (black markers) to track inertial sensors and calculated virtual markers, i.e. joint centers (green markers).

A 12-camera optoelectronic system (Oqus 7+, Qualisys, Göteborg, Sweden) sampled at 100 Hz was used to track the 3D trajectories of 69 reflective markers placed on anatomical landmarks and on clusters. Anatomical markers (14 mm diameter) of the lower body were placed according to the Conventional Gait Model 1.0^18^ (Fig. 1) and the markers of the upper body were placed according to the Plug In Gait model (Vicon Motion Systems, Oxford, UK)^19^. A full description of each marker is reported in Table 1 and in Table 2.

**Table 1:** Anatomical marker trajectories stored in c3d files (n: number of frames recorded at 100 Hz).

Labels	Format	Dim.	Unit	Description
LFHD	Real	n × 3	mm	Left front head trajectories
LBHD	Real	n × 3	mm	Left back head trajectories
RFHD	Real	n × 3	mm	Right front head trajectories
RBHD	Real	n × 3	mm	Right back head trajectories
CLAV	Real	n × 3	mm	Suprasternal notch trajectories
STRN	Real	n × 3	mm	Xiphoid process trajectories
C7	Real	n × 3	mm	7^th^ cervical vertebra trajectories
T10	Real	n × 3	mm	10^th^ thoracic vertebrae trajectories
RBAK	Real	n × 3	mm	Right scapula root spine trajectories
LSHO	Real	n × 3	mm	Left acromial edge trajectories
LELB	Real	n × 3	mm	Left lateral humerus epicondyle trajectories
LWRA	Real	n × 3	mm	Left radius styloid process trajectories
LWRB	Real	n × 3	mm	Left ulnar styloid process trajectories
LFIN	Real	n × 3	mm	Left head of the 3^rd^ metacarpus trajectories
RSHO	Real	n × 3	mm	Right acromial edge trajectories
RELB	Real	n × 3	mm	Right lateral humerus epicondyle trajectories
RWRA	Real	n × 3	mm	Right radius styloid process trajectories
RWRB	Real	n × 3	mm	Right ulnar styloid process trajectories
RFIN	Real	n × 3	mm	Right head of the 3^rd^ metacarpus trajectories
LASI	Real	n × 3	mm	Left anterior-superior iliac spine trajectories
LPSI	Real	n × 3	mm	Left posterior-superior iliac spine trajectories
RASI	Real	n × 3	mm	Right anterior-superior iliac spine trajectories
RPSI	Real	n × 3	mm	Right posterior-superior iliac spine trajectories
SACR*	Real	n × 3	mm	Middle of the PSI distance trajectories
midASIS*	Real	n × 3	mm	Middle of the ASI distance trajectories
LTHI	Real	n × 3	mm	Left lateral femur wand trajectories
LHJC*	Real	n × 3	mm	Left hip joint center trajectories
LKNE	Real	n × 3	mm	Left lateral femoral epicondyle trajectories
LKJC*	Real	n × 3	mm	Left knee joint center trajectories
LTIB	Real	n × 3	mm	Left lateral tibia wand trajectories
LANK	Real	n × 3	mm	Left lateral tibial malleolus trajectories
LAJC*	Real	n × 3	mm	Left ankle joint center trajectories
LTOE	Real	n × 3	mm	Left 2^nd^ metatarsal calcaneus trajectories
LFMH	Real	n × 3	mm	Left 1^st^ metatarsal head trajectories
LHEE	Real	n × 3	mm	Left posterior calcaneus trajectories
RTHI	Real	n × 3	mm	Right lateral femur wand trajectories
RHJC*	Real	n × 3	mm	Right hip joint center trajectories
RKNE	Real	n × 3	mm	Right lateral femoral epicondyle trajectories
RKJC*	Real	n × 3	mm	Right knee joint center trajectories
RTIB	Real	n × 3	mm	Right lateral tibia wand trajectories
RANK	Real	n × 3	mm	Right lateral tibial malleolus trajectories
RAJC*	Real	n × 3	mm	Right ankle joint center trajectories
RTOE	Real	n × 3	mm	Right 2^nd^ metatarsal head trajectories
RFMH	Real	n × 3	mm	Right 1^st^ metatarsal head trajectories
RHEE	Real	n × 3	mm	Right posterior calcaneus trajectories

*Calculated markers.

**Table 2:** Cluster marker trajectories stored in c3d files (n: number of frames recorded at 100 Hz).

Labels	Format	Dim.	Unit	Description
TR1^†^	Real	n × 3	mm	1^st^ trunk cluster marker trajectories
TR2^†^	Real	n × 3	mm	2^nd^ trunk cluster marker trajectories
TR3^†^	Real	n × 3	mm	3^rd^ trunk cluster marker trajectories
TR4^†^	Real	n × 3	mm	4^th^ trunk cluster marker trajectories
SA1^†^	Real	n × 3	mm	1^st^ pelvis cluster marker trajectories
SA2^†^	Real	n × 3	mm	2^nd^ pelvis cluster marker trajectories
SA3^†^	Real	n × 3	mm	3^rd^ pelvis cluster marker trajectories
SA4^†^	Real	n × 3	mm	4^th^ pelvis cluster marker trajectories
LT1^†^	Real	n × 3	mm	1^st^ left thigh cluster marker trajectories
LT2^†^	Real	n × 3	mm	2^nd^ left thigh cluster marker trajectories
LT3^†^	Real	n × 3	mm	3^rd^ left thigh cluster marker trajectories
LT4^†^	Real	n × 3	mm	4^th^ left thigh cluster marker trajectories
RT1^†^	Real	n × 3	mm	1^st^ right thigh cluster marker trajectories
RT2^†^	Real	n × 3	mm	2^nd^ right thigh cluster marker trajectories
RT3^†^	Real	n × 3	mm	3^rd^ right thigh cluster marker trajectories
RT4^†^	Real	n × 3	mm	4^th^ right thigh cluster marker trajectories
LS1^†^	Real	n × 3	mm	1^st^ left shank cluster marker trajectories
LS2^†^	Real	n × 3	mm	2^nd^ left shank cluster marker trajectories
LS3^†^	Real	n × 3	mm	3^rd^ left shank cluster marker trajectories
LS4^†^	Real	n × 3	mm	4^th^ left shank cluster marker trajectories
RS1^†^	Real	n × 3	mm	1^st^ right shank cluster marker trajectories
RS2^†^	Real	n × 3	mm	2^nd^ right shank cluster marker trajectories
RS3^†^	Real	n × 3	mm	3^rd^ right shank cluster marker trajectories
RS4^†^	Real	n × 3	mm	4^th^ right shank cluster marker trajectories
LF1^†^	Real	n × 3	mm	1^st^ left foot cluster marker trajectories
LF2^†^	Real	n × 3	mm	2^nd^ left foot cluster marker trajectories
LF3^†^	Real	n × 3	mm	3^rd^ left foot cluster marker trajectories
LF4^†^	Real	n × 3	mm	4^th^ left foot cluster marker trajectories
RF1^†^	Real	n × 3	mm	1^st^ right foot cluster marker trajectories
RF2^†^	Real	n × 3	mm	2^nd^ right foot cluster marker trajectories
RF3^†^	Real	n × 3	mm	3^rd^ right foot cluster marker trajectories
RF4^†^	Real	n × 3	mm	4^th^ right foot cluster marker trajectories

^†^Refer to Fig. 1 to locate each of the four markers per cluster.

Three force plates sampled at 1000 Hz (AMTI Accugait, Watertown, MA, USA) were used to record 3D ground reaction forces and moments. Available ground reaction forces and moments across participants and trials are accesible in Table 3. A description of force plate data is reported in Table 4.

**Table 3:** Available ground reaction forces and moments across participants and trials (NA: trial not available).

Trial files for each walking task										
Participant Id	P01_S01	P02_S01	P03_S01	P04_S01	P05_S01	P06_S01	P07_S01	P08_S01	P09_S01	P10_S01
**Task**										
Gait_01	XRX	XXX	RXX	XXX	XXR	RXX	XXR	LXX	RXX	XRX
Gait_02	XXX	XXL	LXX	XRX	LXX	XXR	RXX	XXL	XLX	XLX
Gait_03	LXX	XLX	XRX	LXX	XRX	RXX	LXX	LXX	XLX	LXX
Gait_04	XXX	RLX	XLX	LXX	XRX	XXR	XXX	XXL	XXL	RXX
Gait_05	RXX	XRX	XLX	XXX	XXX	RXX	RXL	LXX	XXR	XLX
Gait_06	XXL	XXL	XLX	XLX	LXX	XXR	RXL	XXL	XXL	LXX
Gait_07	XXL	XRX	XXX	XRX	XRX	RXL	RXL	LXX	XXR	XXX
Gait_08	XXL	XLX	LXX	XRX	LXX	LXX	RXL	XXR	RXX	XXX
Gait_09	XXR	XRX	NA	XXR	XXL	RXL	NA	NA	XXL	XLX
Gait_10	RXX	XLX	NA	XXX	XRX	LXX	NA	NA	XXR	XXX
Gait_11	NA	NA	NA	XXX	LXX	NA	NA	NA	XXL	XLX
Gait_12	NA	NA	NA	XXX	XXX	NA	NA	NA	XXR	XLX
SlowGait_01	XXX	XXX	XRL	RLX	XLR	XLR	XXR	LRX	LXX	XXR
SlowGait_02	XLR	XXX	XXR	RXX	XXR	XXX	XXX	XXX	RLR	XXX
FastGait_01	XXX	XXX	XXL	XXX	XXX	LXR	XRX	XXX	LXX	NA
FastGait_02	XRX	XXX	XXR	RXL	XXR	RXX	XXL	XXX	XXX	NA
2minWalk_01	XXX	XXR	NA	XXL	LXX	XXX	RXX	XLX	XXX	RXX
2minWalk_02	LXX	LXX	NA	XXX	XRX	XXX	LXX	LXX	XXX	XXX
2minWalk_03	XXX	NA	NA	XXL	XXX	LXX	XXL	RXX	XXX	XXX
2minWalk_04	XLX	NA	NA	NA	NA	NA	NA	NA	NA	NA
Running_01	XXX	XXL	LXR	XXL	XXL	RXL	LXR	XLX	RXL	XXL
Running_02	NA	XRX	XXX	XXX	XLX	LXR	RXL	XXX	XXL	RXX

The first letter corresponds to the force plate (FP) number 1, the second to the FP number 2 and the last one to the FP number 3. The letter L corresponds to the left foot, the letter R for the right foot and the letter X for no correct data available (i.e. XXL with no correct data available for FP1 and FP2 and a left foot on FP3; LXR with a left foot on FP1, no correct data available on FP2 and a right foot on FP3; etc.).

**Table 4:** Force plates data stored in c3d files; Data provided in the optoelectronic coordinate system; p: number of frames recorded at 1000 Hz.

Labels	Format	Dim.	Unit	Description
Fx1	Real	p × 3	N	Force applied by the foot on platform 1 component X
Fy1	Real	p × 3	N	Force applied by the foot on platform 1 component Y
Fz1	Real	p × 3	N	Force applied by the foot on platform 1 component Z
Mx1	Real	p × 3	N.mm	Moment applied by the foot on platform 1 component X
My1	Real	p × 3	N.mm	Moment applied by the foot on platform 1 component Y
Mz1	Real	P × 3	N.mm	Moment applied by the foot on platform 1 component Z
Fx2	Real	p × 3	N	Force applied by the foot on platform 2 component X
Fy2	Real	p × 3	N	Force applied by the foot on platform 2 component Y
Fz2	Real	p × 3	N	Force applied by the foot on platform 2 component Z
Mx2	Real	p × 3	N.mm	Moment applied by the foot on platform 2 component X
My2	Real	p × 3	N.mm	Moment applied by the foot on platform 2 component Y
Mz2	Real	p × 3	N.mm	Moment applied by the foot on platform 2 component Z
Fx3	Real	p × 3	N	Force applied by the foot on platform 3 component X
Fy3	Real	p × 3	N	Force applied by the foot on platform 3 component Y
Fz3	Real	p × 3	N	Force applied by the foot on platform 3 component Z
Mx3	Real	p × 3	N.mm	Moment applied by the foot on platform 3 component X
My3	Real	p × 3	N.mm	Moment applied by the foot on platform 3 component Y
Mz3	Real	p × 3	N.mm	Moment applied by the foot on platform 3 component Z

**Fig. 2:**
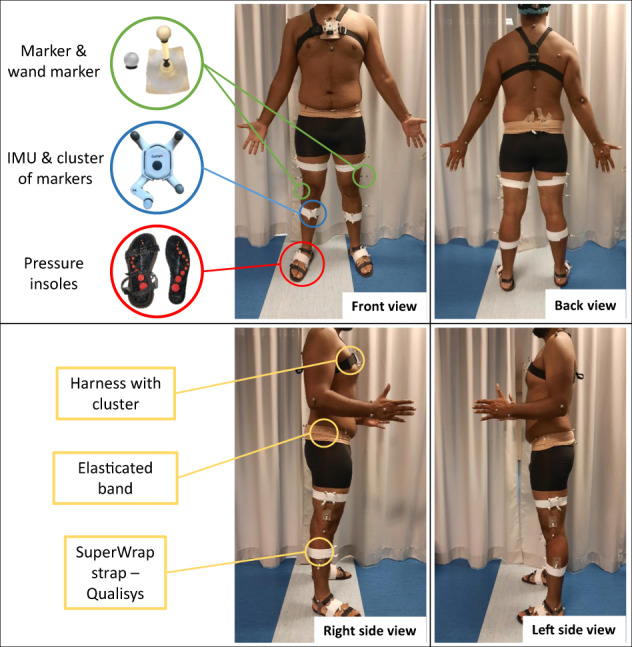
Full setup on a participant, including systems used during the measurement and fixing tools.

**Fig. 3:**
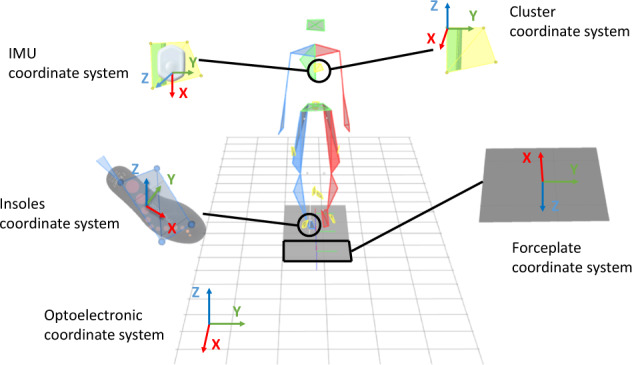
Definition of the coordinate systems of each device used during the measurement.

Eight IMUs (Physilog6S, GaitUp, Renens, Switzerland) sampled at 256 Hz were strapped (SuperWrap, Qualisys, Göteborg, Sweden) on feet, shanks, thighs, pelvis and trunk (Figs. 1, 2). They recorded 3D linear accelerations with a range of ±16 g, 3D angular velocities with a range of ±2000 °/s, 3D magnetic field intensity with a range of ±50 mT, and the barometric altitude from 260 to 1260 hPa. A short description of IMUs data is presented in Table 5. The IMUs were switched on about 10 minutes before the beginning of the recordings, levelled horizontally, and all aligned. The measurement of the IMUs at rest can be found in the raw data (bin file). It is therefore possible for each user to develop its own algorithms. The “master” sensor was always switched on last, in order to have the same routine for all measurements and to have approximately the same switch-on delay. Each IMU was placed on a 3D printed cluster of four markers (9.5 mm diameter) to track its position and orientation in the global coordinate system defined by the optoelectronic system (Fig. 3). Then, IMUs were switched off after the end of recordings under the same conditions as for the switch on.

**Table 5:** Inertial sensor (IMU) data stored in bin files; m: number of frames recorded at 256 Hz.

Labels	Format	Dim.	Unit	Description
Accel1 X	Real	m × 1	g	Linear acceleration component X
Accel1 Y	Real	m × 1	g	Linear acceleration component Y
Accel1 Z	Real	m × 1	g	Linear acceleration component Z
Gyro1 X	Real	m × 1	deg.s^−1^	Angular velocity component X
Gyro1 Y	Real	m × 1	deg.s^−1^	Angular velocity component Y
Gyro1 Z	Real	m × 1	deg.s^−1^	Angular velocity component Z
Mag1 X	Real	m × 1	Gauss	Magnetic field component X
Mag1 Y	Real	m × 1	Gauss	Magnetic field component Y
Mag1 Z	Real	m × 1	Gauss	Magnetic field component Z
Quat1 W	Real	m × 1	/	Scalar part of the quaternion
Quat1 X	Real	m × 1	/	Vector part of the quaternion
Quat1 Y	Real	m × 1	/	Vector part of the quaternion
Quat1 Z	Real	m × 1	/	Vector part of the quaternion

**Fig. 4:**
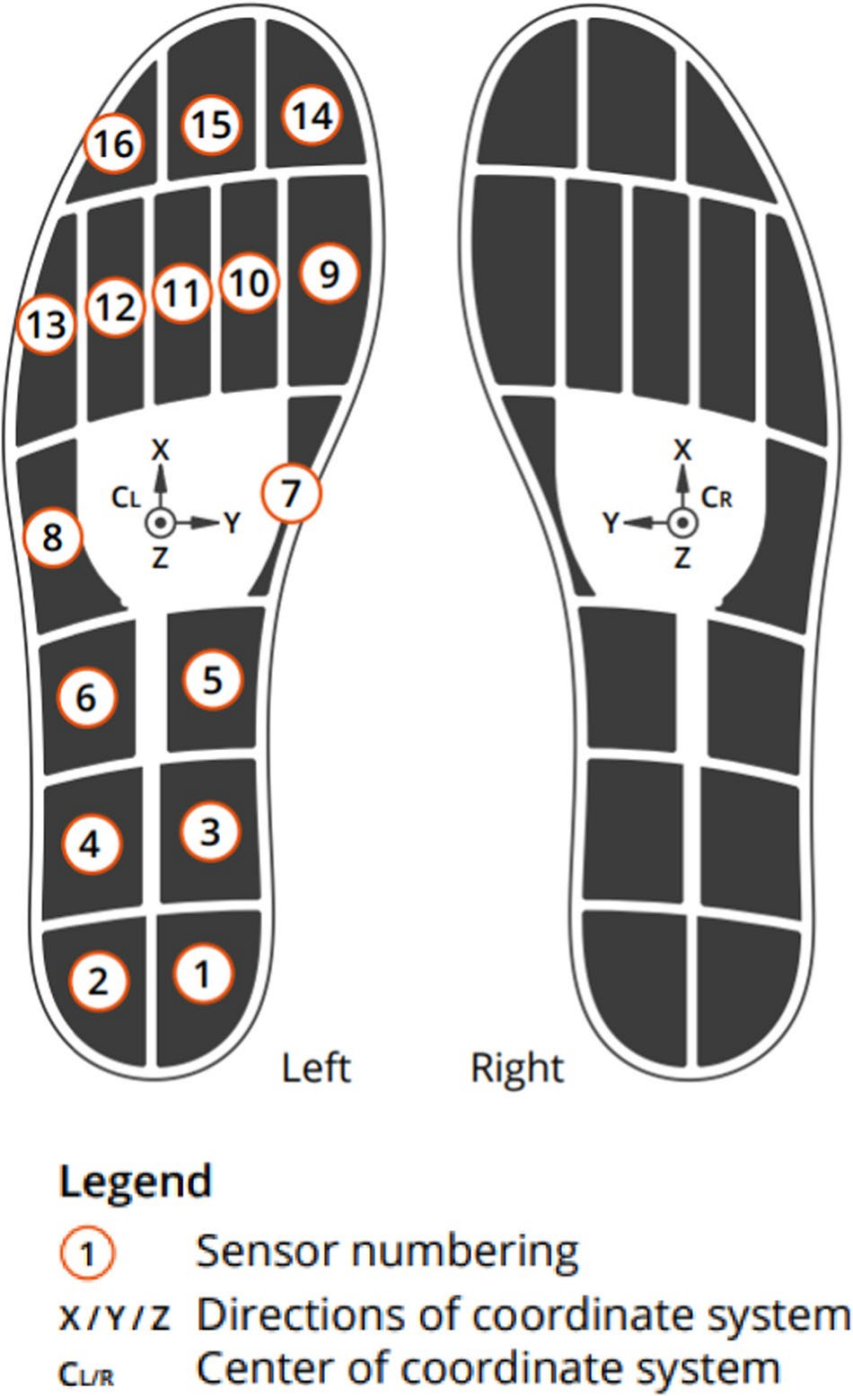
Sensor insoles outline and plantar sensor positions. Adapted from Moticon Science Sensor Insole specification.

Two insoles (Insole3, Moticon ReGo AG, Munich, Germany) sampled at 100 Hz were used to measure feet plantar pressures. These insoles were composed of 16 pressure sensors, a 3D accelerometer and a 3D gyroscope, as described in Table 6 and Fig. 4. The total area covered by pressure sensors ranged from 7.6 mm^2^ to 16.5 mm^2^ depending on sensor insoles (Fig. 4). They were switched on just before the beginning of the recordings (after the participant preparation) and were switched off after the end of recordings and data transfer to the computer.

**Table 6:** Pressure insole data stored txt files; q: number of frames recorded at 100 Hz.

Labels	Format	Dim.	Unit	Description
Left pressure	Real	q × 16	N.cm^−2^	Left pressure for the 16 pressure sensors
Left acceleration X	Real	t × 1	g	Linear acceleration for the left insole component X
Left acceleration Y	Real	t × 1	g	Linear acceleration for the left insole component Y
Left acceleration Z	Real	t × 1	g	Linear acceleration for the left insole component Z
Left angular X	Real	t × 1	deg.s^−1^	Angular velocity for the left insole component X
Left angular Y	Real	t × 1	deg.s^−1^	Angular velocity for the left insole component Y
Left angular Z	Real	t × 1	deg.s^−1^	Angular velocity for the left insole component Z
Left total force	Real	q × 1	N	Total force for the left insole
Left center of pressure X	Real	q × 1	%	Center of pressure for the left insole component X
Left center of pressure Y	Real	q × 1	%	Center of pressure for the left insole component Y
Right pressure	Real	q × 1	N.cm^−2^	Right pressure for the 16 pressure sensors
Right acceleration X	Real	t × 1	g	Linear acceleration for the right insole component X
Right acceleration Y	Real	t × 1	g	Linear acceleration for the right insole component Y
Right acceleration Z	Real	t × 1	g	Linear acceleration for the right insole component Z
Right angular X	Real	t × 1	deg.s^−1^	Angular velocity for the right insole component X
Right angular Y	Real	t × 1	deg.s^−1^	Angular velocity for the right insole component Y
Right angular Z	Real	t × 1	deg.s^−1^	Angular velocity for the right insole component Z
Right total force	Real	q × 1	N	Total force for the right insole
Right center of pressure X	Real	q × 1	%	Center of pressure for the right insole component X
Right center of pressure Y	Real	q × 1	%	Center of pressure for the right insole component Y

### Procedure

For each participant, the entire data collection was performed in a single session which lasted approximately one hour. All sessions were managed by two operators, one was experienced in placing the anatomical markers and the other performed data recordings. The following procedure was adopted:

#### Calibration of the systems

The calibration of the motion capture system was performed following the instructions available in the manufacturer’s documentation, including the definition of the global coordinate system, the dynamic calibration of the cameras, and the zeroing of the force plates. The calibration of the pressure insoles was also performed following the instructions of the manufacturer’s documentation, including the zeroing (i.e. the automatic zeroing mode is based on algorithms continuously checking sensor zero levels and compensates for sensor offsets and drifts which may occur due to lacing shoes and due to temperature changes. In addition, a manual zeroing routine can be carried out by means of a software function^20^) and the calibration (i.e. the calibration routine is carried out using the app and included very-slow gait and static postures^20^) of the insoles. No calibration was required for the IMUs following the manufacturer’s recommendations.

#### Introduction to the participant

The operators introduced the laboratory, and briefly explained the conduct of the session, and the material used.

#### Preparation of the participant

The participant was asked to change clothes to tight-fitting clothes or underwear, and was asked to be barefoot. An operator collected the participant’s anthropometric data (i.e. height, body mass, as well as pelvic, knees and ankles width). The participant was then equipped with cutaneous reflective markers attached with double-sided adhesive tape, IMUs placed in marker clusters and fixed with straps, and sensor insoles placed in sandals (participants had to walk with shoes) (Fig. 2).

#### Synchronization record

Systems’ synchronization tasks (i.e. IMU-to-optoelectronic and insole-to-IMU synchronizations) were performed at the beginning and at the end of each session since no hardware synchronization was available. IMU-to-optoelectronic synchronization task consisted of inducing a vertical acceleration on a rod equipped with an IMU and two reflective markers. Insole-to-IMU synchronization task consisted of a jump performed by the participant equipped with IMUs and insoles. The alignment of the systems’ timestamps was performed in post-processing using the recorded data.

#### Static trial

A 10-second recording was performed with the participant standing upright (T-pose) on the force plate in the middle of the walkway with straight legs, and parallel feet hip-width apart (Supplementary Table 1). This position allowed a recording with all the markers to reconstruct marker trajectories when there were marker trajectory gaps by rigid transformation^21^. The proper propostion of the subject was carefully controlled by the experimenter (Supplementary Table 1) before starting the acquisition since the task was to be used for IMU-to-segment calibration^16,22,23^. The verbal instruction was: *“Spread your arms slightly and stay in a static position for 10* *seconds without moving.”*

#### Walking trials

The participant was asked to walk back and forth on the 10-m walkway at three different speeds: comfortable speed, slow speed and fast speed. No instruction was given about the force plates to ensure the most natural gait. A minimum of 8 trials were recorded for each subject for the comfortable speed to ensure a minimum of 7 validated force plate data (left and right side taken together), and 2 trials were recorded for the two other speeds whatever the force plate recordings. Details of available force plate data for each participant and for each task are reported in Table 1. The verbal instruction for the comfortable speed was: *“Walk naturally to the other end of the walkway at a comfortable speed.”; The v*erbal instruction for the slow speed was: *“Walk more slowly than before to the other end of the walkway.”; The v*erbal instruction for the fast speed was: *“Walk quickly to the other end of the walkway but do not run.”* The instructions given to the participants are detailed in Supplementary Table 1.

#### Running trials

The participant was asked to run at a spontaneous speed without sprinting in order to avoid too many marker occultations. Two trials were recorded whatever the force plate recordings. The verbal instruction was: *“Run to the other end of the walkway without sprinting.”* (Supplementary Table 1).

#### Calibration trials

Five different movements, being 5 independent trials stored in separate files, were performed by the participant: 1) 5 squats with a medium flexion and standing on the heels at the end of knees extension, 2) 5 hip abductions and adductions on both sides consecutively, 3) 5 hip/knee/ankle flexions on both sides consecutively, 4) 5 hip rotations on both sides consecutively, and 5) 5 pelvis rotations, 5 pelvis anteversions and retroversions, and 5 pelvis obliquities. Because of the length of all the verbal instructions, these are detailed in Supplementary Table 1. These trials can be used to perform IMU-to-segment calibration^16,22,23^.

#### Timed Up and Go trials

Two Timed Up and Go (TUG) tests^24^ were performed, one with a 3-m walking part and another one with 5-m walking part. The participant started out sitting on a stool, got up, walked 3 or 5 meters, turned around and returned to sit on the stool. No instruction was given to the participant to perform the task, he/she just started with his/her arms at their sides (Supplementary Table 1). The participant was seated on a stool and not a chair to avoid back marker occultations. The verbal instruction was: *“Stand up and walk 3 meters to the cone, turn around and walk back to the stool, turn to sit down and sit down.”*

#### Sit to Stand trials

This task was adapted from the standardized sit to stand test^25^. The participant was asked to stand up from a stool and sit down five consecutive times^26^ as quickly as possible. Participant’s arms were crossed on stomach or chest to avoid marker occultations (Supplementary Table 1). The verbal instruction was: *“Stand up and sit down 5 times as fast as possible.”*

#### Sitting trials

The participant began in a sitting position with an angle of 90° at hip, knee and ankle levels, and with a straight chest. A minimum of five seconds without movement were recorded. Then, the participant was asked to extend his/her legs and tilt his/her chest, and a minimum of five seconds without movement were again recorded. Finally, a passive flexion/extension of the knee and ankle was induced by the operator on both sides consecutively (Supplementary Table 1). The verbal instruction was: *“Keep your arms at your sides and sit still for 5* *seconds (without moving). Then, sit with your legs straight, without moving, for 5* *seconds – only your heels touch the ground.”*

#### 2-minute trials

The participant was asked to walk back and forth along 20 m at comfortable speed. No instruction was given about the force plates to ensure the most natural gait. Since the field of view of the optoelectronic cameras is only 5 m and to avoid having a too long recording, several recordings were started each time the participant entered the cameras’ field of view. Thus, during the 2-minute trial, there were at least three recordings. The verbal instruction was: *“Walk naturally to the other end of the walkway at a comfortable speed, and turn around and go to the other end during 2* *minutes.”*

### Data processing

Three-dimensional marker trajectories and ground reactions were labelled with the Qualisys Tracking Manager software (QTM 2019.3, Qualisys, Göteborg, Sweden). Raw data were exported in c3d file format (https://www.c3d.org) and processed under Matlab (R2019b, The MathWorks, USA) using the Biomechanics Toolkit (BTK)^27^. Marker trajectories processing consisted in an interpolation to fill gaps using a reconstruction based on marker inter-correlations^28^.

Joint centers of the lower limbs and the center of the posterior and anterior iliac spines were calculated and added as virtual markers in each trial file. Hip joint centers were computed with Hara’s regression equations^29^, while other joint centers were calculated with a chord function^30^.

Ground reaction processing consisted in (1) a data zeroing and (2) 3D centre of pressure (CoP) recomputation. The zeroing process was required as some trials showed an offset of the force data, even if a platform reset was performed at the beginning of each session.

The CoP recomputation was required due to incompatibility of the BTK c3d parser and the type of force plate and in particular the AMTI Accugait Type 5 used in this study. A custom Matlab program was used for this purpose. No filtering was applied on both marker trajectories and force plates data.

Gait events (i.e. foot strikes and foot offs) related to slow gait, comfortable gait, fast gait and 2-min trials were automatically detected using a validated algorithm proposed by Zeni *et al*.^31^. Each file containing the events detected has been visually checked and corrected if necessary using the open-source software Mokka (http://biomechanical-toolkit.github.io/). For this process, running and TUG trials were excluded to avoid any detection problems due to marker occultations and turnaround during tests. The three force plates were not used for gait event detection because of the low number of steps. Regarding the insoles, we did not have direct access to the events detected by the software and we did not implemented/validated any code for it. It is still possible to add events manually using for instance the open-source software Mokka.

All data and metadata (description of the data organization provided in the dataset^9^) recorded by the optoelectronic system and force plates were stored in c3d file format (binary files, see Code Availability section for file reading and https://www.c3d.org/) by task and session.

IMUs position and orientation were tracked using the marker clusters to perform calibrations. Indeed, many studies have shown the importance of performing a IMU-to-segment calibration^16,22,23^ to ensure that IMUs are properly aligned with the anatomical axes. This calibration largely influences kinematics computation^22^. Several IMU-to-segment approaches exist; the most common being the functional calibration which consists of aligning the sensors and segments frames based on specific 2D-movements realized by the subject^23^. Thus, the proposed dataset^9^ is composed of several functional tasks performed in different anatomical planes to allow IMU-to-segment calibration (e.g. flexion/extensions, rotations, ab/adductions).

No data processing was performed on IMUs and insoles data. The IMUs data were stored in bin file format (binary files, see Code Availability section for file reading) and the insoles data in txt file format (ASCII files) by session.

Optoelectronic and IMUs data could not be synchronized with a hardware system because the version of IMUs we used did not include this function. Thus, the data were synchronized using the start and end impacts with acceleration peak detection (see Procedure 4. *Synchronization record*). IMUs and insoles data were synchronized using jump events. The optoelectronic and force plates data were not cut. For the IMUs and insoles files, the data were synchronized respectively with peak detection and cross-correlation functions. Then, these data were resampled to 100 Hz (optoelectronic sampling frequency), cropped according to the synchronized timestamps and stored in a csv format (ASCII file) by task and session. These files contain synchronized marker trajectories, linear accelerations and angular velocities of IMUs and all insole data.

All other data such as anthropometric data as well as non-synchronized force plate data, gait events, and IMU barometers are only stored in c3d or bin files. It should be noted that the synchronization procedure precision is approximated at 0.1 second due to the manual detection performed using a custom Matlab program. The difficulty was to be precise for the operator.

Thus, it is strongly recommended that each user resynchronizes the data for their own applications if needed. For this purpose, users can use the raw data present in the data repository.

### Missing data

There was no insole data for Participant 01 because of a delay in the delivery of the devices from the start of the study. During the 2-minute trials of the Participant 03 the Qualisys Tracking Manager software crashed and recordings could not be saved. Finally, for the Participant 05, the left thigh (LT) IMU sensor stopped during the measurement and no data were recorded.

## Data Records

### Data description

All recorded data are described in detail in Excel files available in the dataset^9^. Each optoelectronic, IMUs, and insole data have their own Excel file with a detailed description of the acquired data (e.g. for the IMUs, the parameters of each electronic sensor are described as well as the location of the “master” sensor and the synchronization information data between the sensor set). Moreover, in the optoelectronic Excel file, a complete list of tasks performed or not by the participants as well as associated comments (in case of errors or problems occurred during data acquisition and post-processing) are given. Finally, each Excel file contains a “Description” sheet that summarizes the content of the other sheets present in the file.

All data files are available online on a *Yareta* database^9^: 10.26037/yareta:xkxgaw6ewjdhfntdhtj7upepxy.

Data are organized by participant folder (PXX_SYY, P: for *Participant*, S: for *Session*) and each folder contains two sub-folders:

- RAW_DATA
  - One .c3d file per trial recorded during the session
  - Eight .bin files corresponding to the IMUs data recorded during the session
  - One .txt file corresponding to the insole data recorded during the session

- SYNC_DATA
  - One .csv file per trial with all the optoelectronic, IMUs and insole synchronized data recorded during the session

C3D trial files are referenced in our dataset^9^ as PXX_SYY_[Trial type]_[Trial number].c3d, with the following correspondence:

- P: for *Participant*
- XX: participant number (e.g. 01)
- S: for *Session*
- YY: session number (e.g. 01)
- [Trial type]: task performed (Table 7)

**Table 7:** Description of trial tasks.

Trial type	Number of trials by participant [min - max]	Description
Synchronization	[2–5]	Synchronization tasks used to synchronize the different systems
Static	1	Static standing posture
Sitting	1	Static sitting posture, straight legs static sitting posture and passive knee and ankle flexion/extension
CalibrationTask	5	Movements in different anatomical planes (separated in 5 trials)
Gait	[8–12]	Gait at comfortable walking speed
SlowGait	2	Gait at slower speed than comfortable walking speed
FastGait	[0–2]	Gait at faster speed than comfortable walking speed
Running	[1–2]	Jogging
2minWalk	[0–4]	2 min walk without stop at a comfortable speed
SitToStand	[0–2]	5 consecutive stand-ups and sit-downs
TUG	2	3-m and 5-m Timed Up and Go
- [Trial number]: trial number (e.g. 01)

IMUs are referenced as PXX_SYY_ZZ_Inertial_sensor.bin, with:

- ZZ: sensor name including TR: torso/SA: pelvis/RT: right thigh/RS: right shank/RF: right foot/LT: left thigh/LS: left shank/LF: left foot

Insoles data are referenced as PXX_SYY_Sensor_insoles.txt.

Synchronized data are referenced with the same name as c3d files but with the file extension .csv.

## Technical Validation

### Calibration of the optoelectronic system

The optoelectronic system was calibrated before each session following the instruction available in the manufacturer’s documentation. The standard deviation of the calibration tool length for all the sessions was on average 1.5 mm for a calibrated volume of 5 × 2 × 2 m and the average residuals (i.e. the minimum distance between a 2D marker ray and its corresponding 3D point) of the markers were below 2.5 mm. This value of 1.5 mm could be reduced for a smaller volume, but for the volume used in this study, it was not possible to define a focal length allowing a sharpness on the whole length of 5 m.

### 3D trajectories of reflective markers

For all dynamic trials of all sessions, the 3D marker trajectories were fill gapped and 0% of gap is thus present in the reported trajectories, except for synchronization tasks where a gap may be present at the impact. Average residuals of the markers were below 1.3 mm. These data were not filtered.

### Inertial sensors

No calibration task was recommended by the manufacturer’s documentation, as the IMUs have been calibrated in factory. The sensor noises are as follows:

- Accelerometer: average standard deviation <7 mg (milli g-force)
- Gyroscope: average standard deviation <1.3 dps (degree per second)
- Magnetometer: average standard deviation <10 mG (milli-Gauss) for X- and Y-axes, <15 mG for Z-axis
- Barometer: average standard deviation <0.06 mBar (after low frequency variation removal)

### Sensor insoles

Sensor insoles were calibrated for each participant before the session according to the instruction given by the manufacturer. A zeroing of each insole was performed before the start of the acquisition as well as a calibration sequence consisting of a slow walking, a standing still and body weight shifts. Temporal drift, i.e. the total drift of the timing data over time since the start of the measurement, has been estimated by the manufacturer to be less than 1%.

## Limitations

The main limitation of using this data set is the small number of participants. Indeed, only 10 participants were included and three of them had missing data.

Another limitation is the synchronization of the systems. Because of the lack of synchronization accuracy (0.1 s) and data cropping, the authors suggest that users use the synchronized data with caution or develop their own synchronization algorithms.

In addition, no information was given by the manufacturer of the IMUs, either for sensor calibration or gyro zeroing. The gains and offsets were therefore not modified during the measurement.

The use of different acquisition frequencies between the systems (256 Hz for IMUs and 100 Hz for optoelectronics) may lead to errors during resampling. The value of 100 Hz for the optoelectronic data was chosen to keep some consistency between the acquired data and the existing database of the laboratory. Regarding the 256 Hz frequency for the IMUs, it was chosen to record high speed running movements (sprint tasks). However, these tasks are not present in the dataset because the optoelectronic data were not usable.

## Usage Notes

Optoelectronic data and metadata stored in c3d files can be directly read using the open-source motion analyzer software Mokka (http://biomechanical-toolkit.github.io/). It is also possible to read these data in a scripting software (e.g. Matlab) using a c3d parser like Biomechanics ToolKit (BTK) (http://biomechanical-toolkit.github.io/). Concerning IMUs data, the manufacturer provides a software to read the sensor signals stored in bin files (Research Toolkit for Physilog6S, https://research.gaitup.com/support/#1610537099987-3-1). A Matlab toolkit is also available on their website (https://physilog.com/). The raw data of sensor insoles can be read with any text editor.

## Supplementary information

Supplementary Table 1

## Data Availability

Matlab codes used to preprocess data, compute joint centers and identify gait events are shared in open access through dedicated gitlab repositories (respectively: https://gitlab.unige.ch/KLab/preprocessing_toolbox, https://gitlab.unige.ch/KLab/fusion_biplane_xrays_motion_capture and https://gitlab.unige.ch/KLab/gev). The Biomechanics Toolkit (BTK) is freely available on the following repository: https://github.com/Biomechanical-ToolKit/BTKCore. The IMUs data reader is freely available on the following website: https://physilog.com/).
