# Supplementary material for: A dataset of asymptomatic human gait and movements obtained from markers, IMUs, insoles and force plates
Source: Sci Data. 2023 Mar 30;10:180. doi: 10.1038/s41597-023-02077-3 (PMC10063557; doi:10.1038/s41597-023-02077-3)
Supplement: Supplementary file 1 — Supplementary Table 1 [file 41597_2023_2077_MOESM1_ESM.docx]

| Supplementary dataTitle A dataset of asymptomatic human gait and movements obtained from markers, IMUs, insoles and force plates. Authors Gautier Grouvel^1^, Lena Carcreff^1^, Florent Moissenet^1,2^, Stéphane Armand^1^  **Affiliations**  1. Kinesiology Laboratory, Geneva University Hospitals and University of Geneva, Geneva, Switzerland  2. Biomechanics Laboratory, Geneva University Hospitals and University of Geneva, Geneva, Switzerland  **Corresponding author(s)**:  Gautier Grouvel, gautier.grouvel@unige.ch Supplementary data **Supplementary Table 1** – Description of all the tasks performed during the measurement, as well as the position controls performed by the operator and the instructions given to the participants. |
| --- |

| **Tasks** | **Positions** | **Position controls** | **Instructions** |
| --- | --- | --- | --- |
| **Static** | **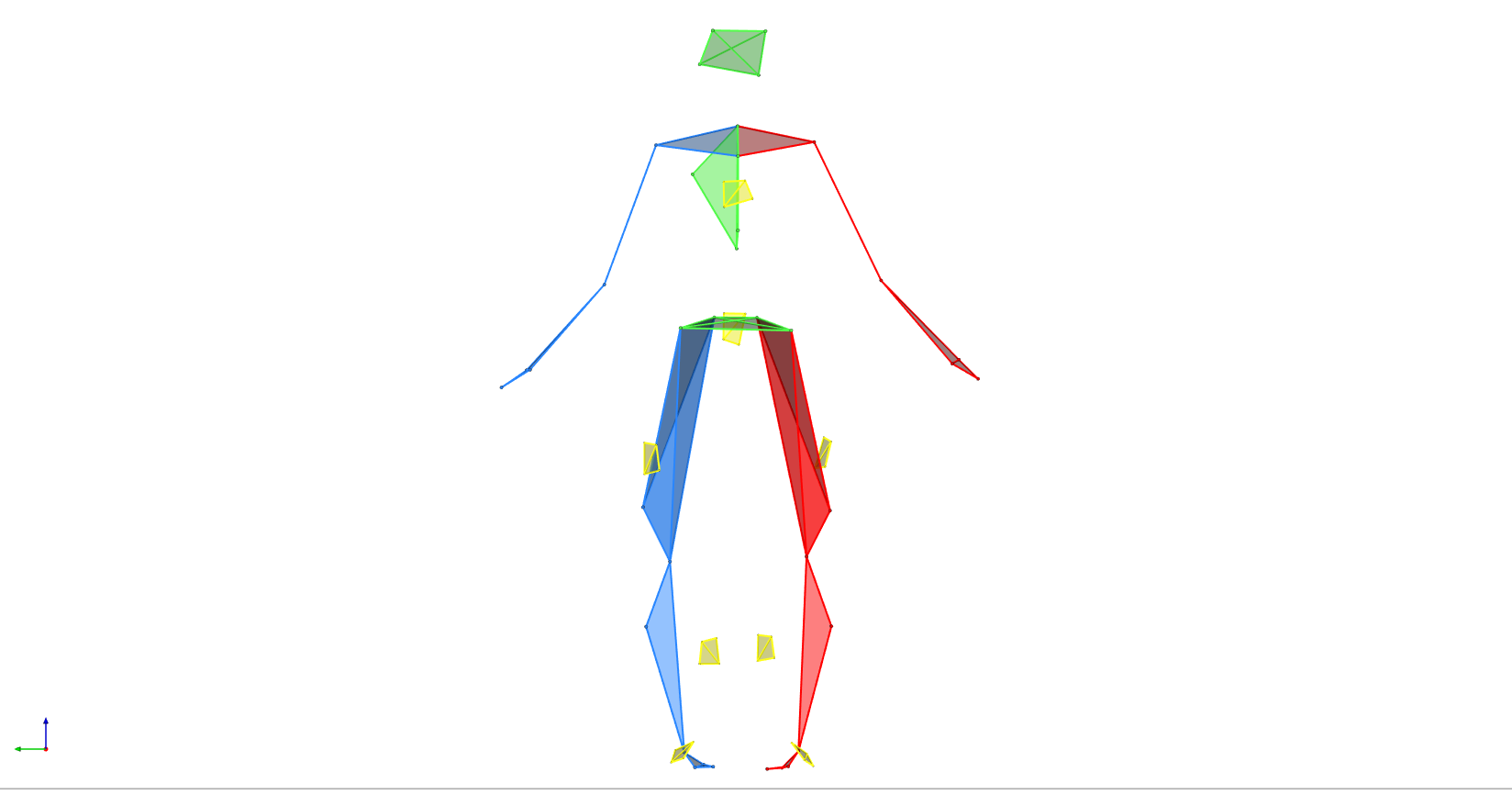** | - Straight legs - Feet parallel - Feet aligned with the hip joint - 10 seconds | *Spread your arms slightly and stay in a static position for 10 seconds without moving* |
| **Sitting** | 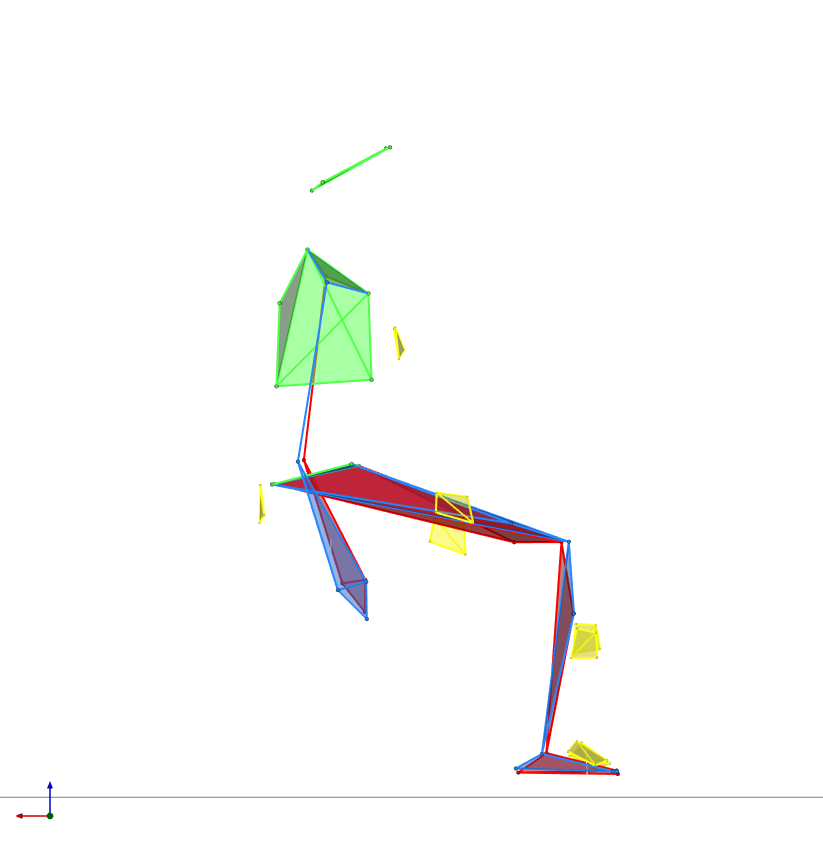 | - Knees and ankles at 90° - Joint aligned in sagittal plane - Arms at the side of the body - 5 seconds | *Keep your arms at your sides and sit still for 5 seconds (without moving)* |
| **Sitting straight legs** | 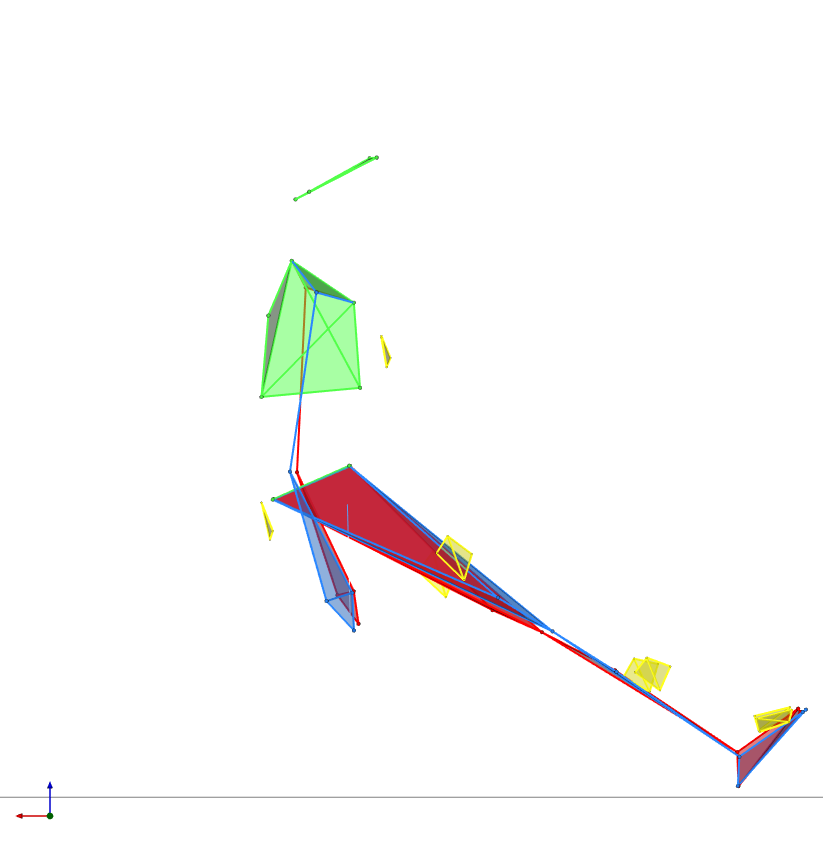 | - Straight legs - Ankles at 90° - Legs aligned with the hip joint - Arms behind the back to hold on - 5 seconds | *Sit with your legs straight, without moving, for 5 seconds - only your heels touch the ground* |
| **Passive movements** | 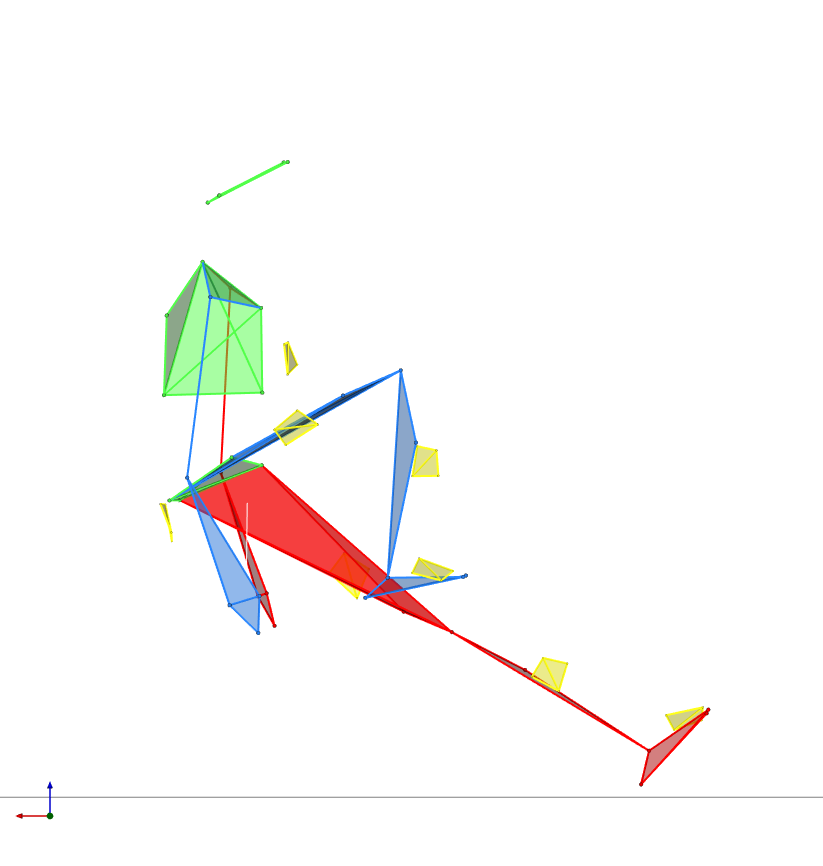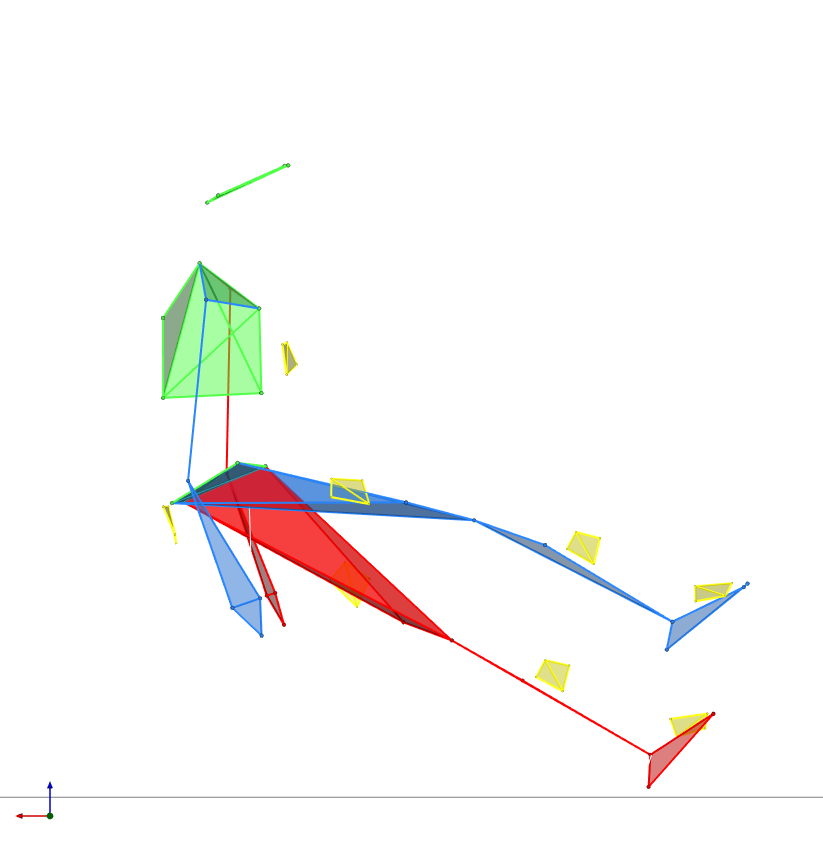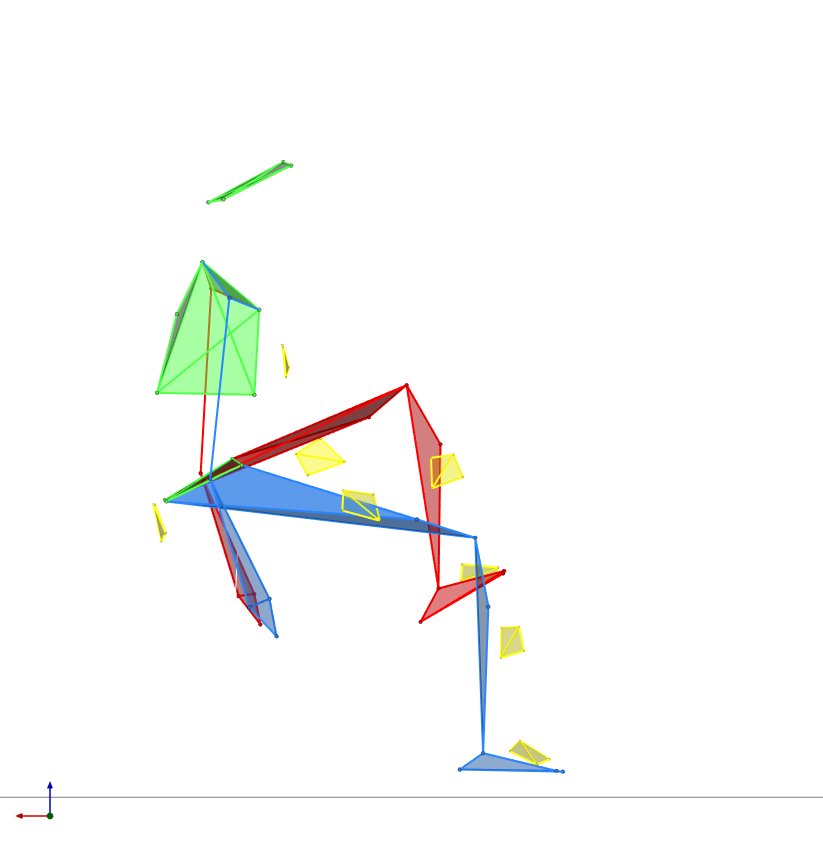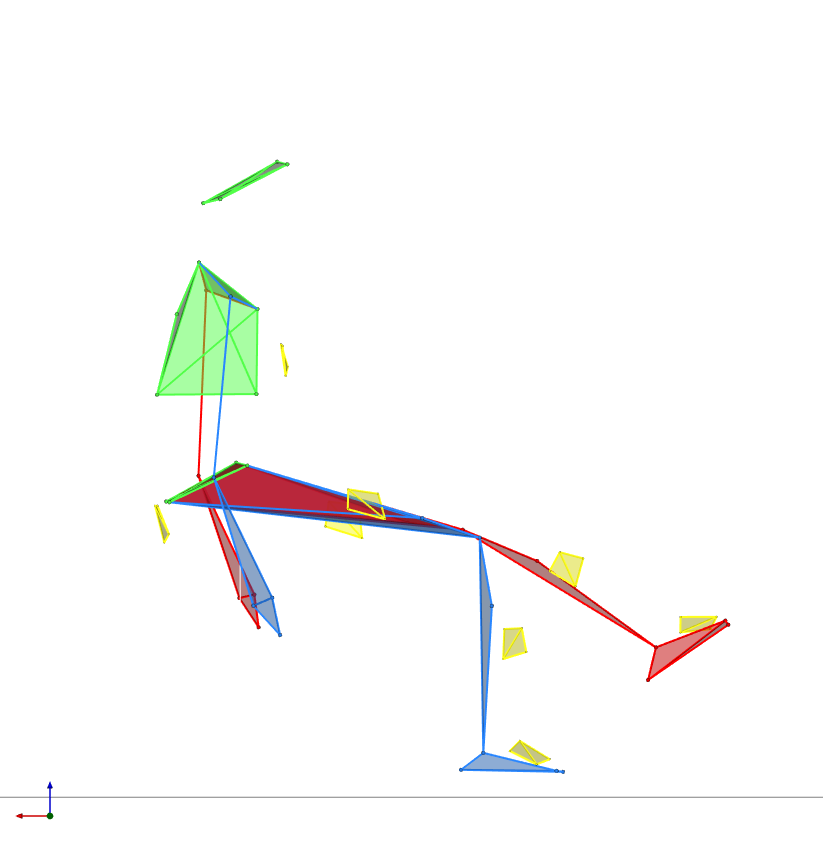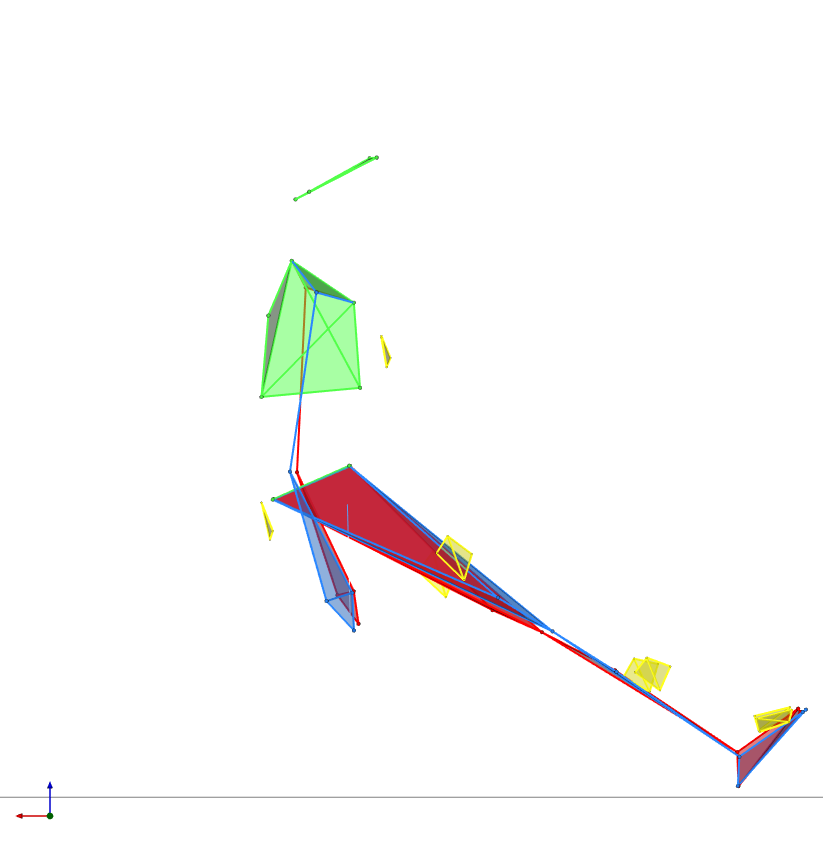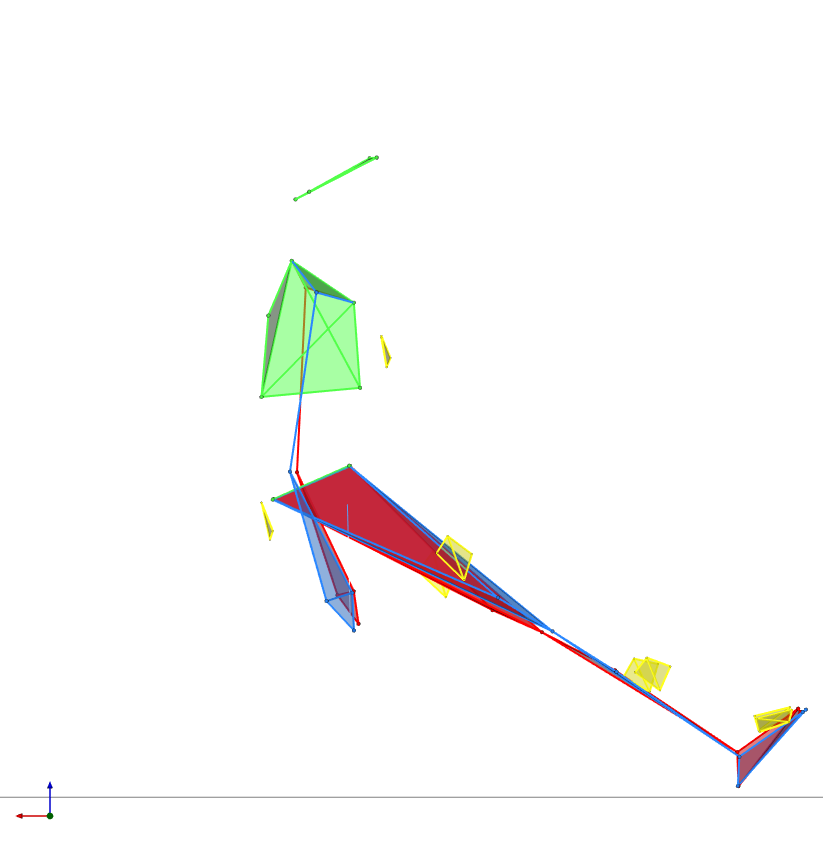 | - Passive flexion/extension movement of the hip, knee and ankle - Performed by the investigator - Joint aligned in sagittal plane - 5 repetitions on the right, then 5 repetitions on the left side | |
| **Squats** | 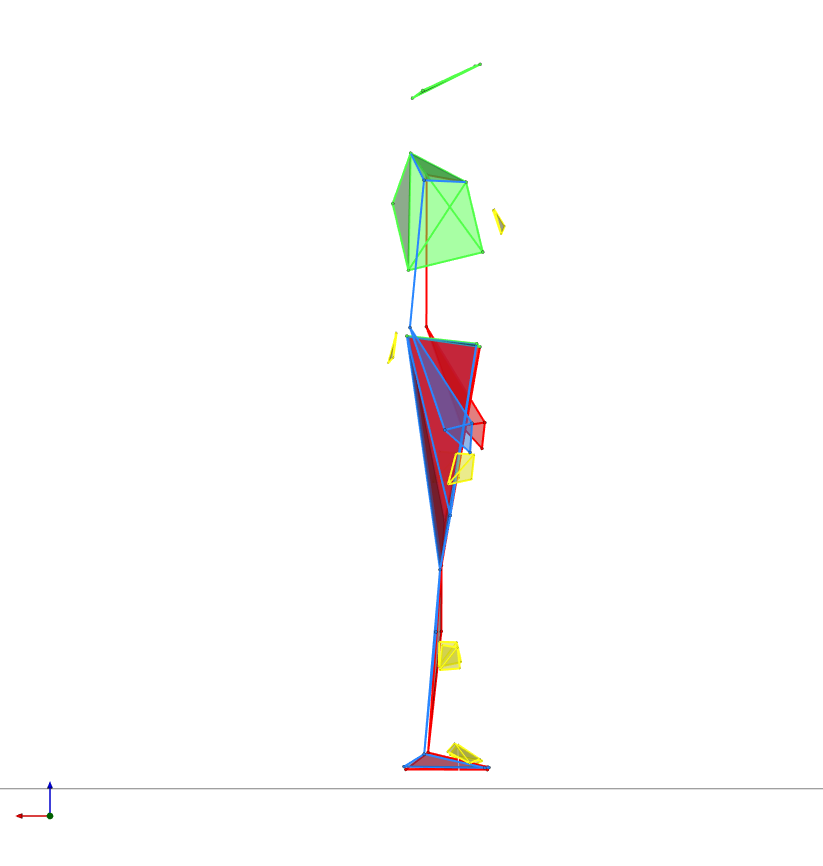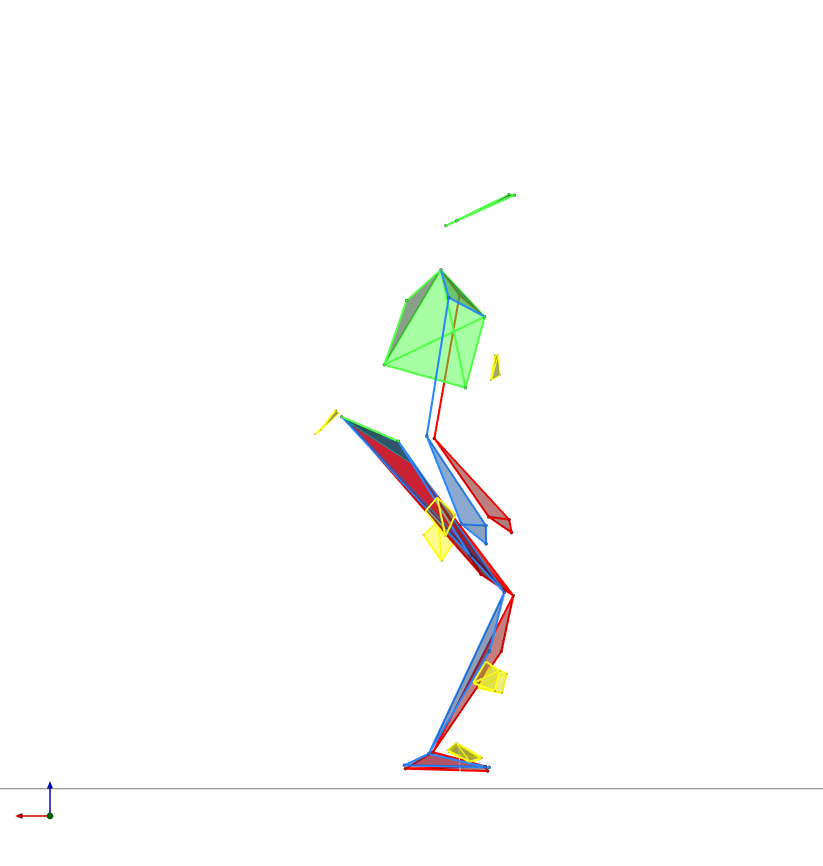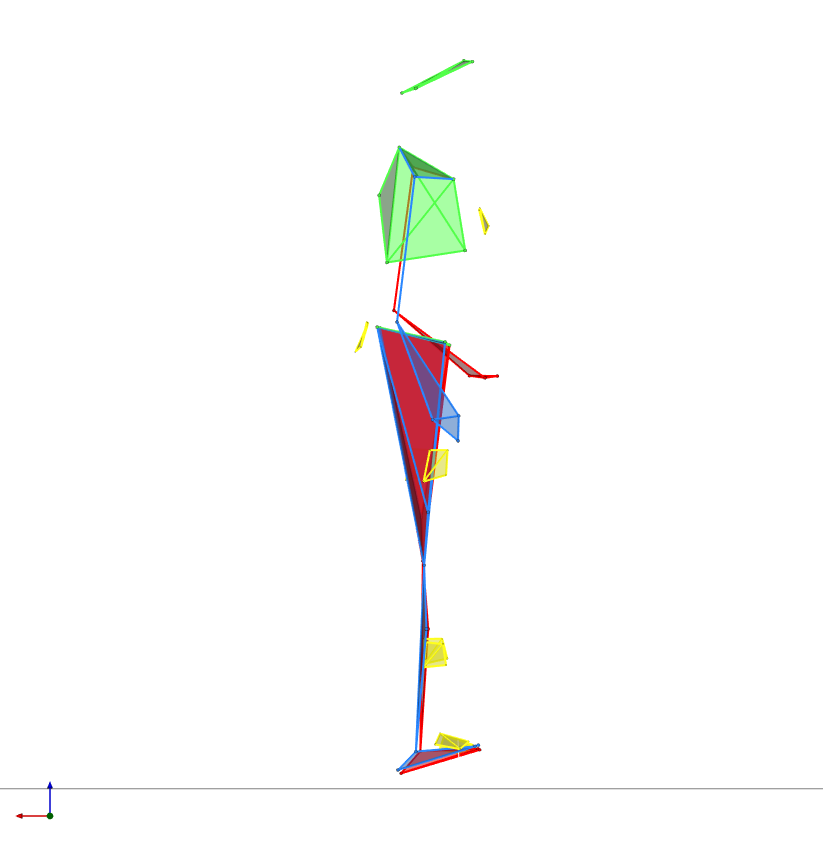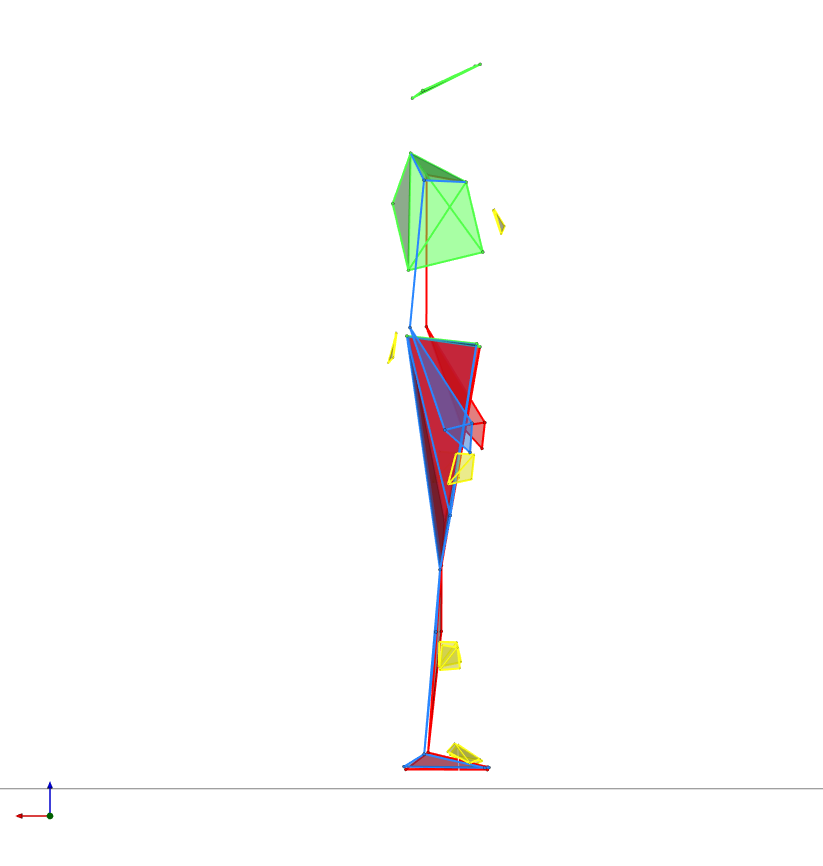 | - Arms at the side of the body - Feet aligned with the hip joint - Half squats - Get om the heels after the squat - 5 repetitions | *Get into a static position, arms by your side, bend your knees, come up and stand on your heels, 5 repetitions* |
| **Hip / Knee / Ankle flexion** | 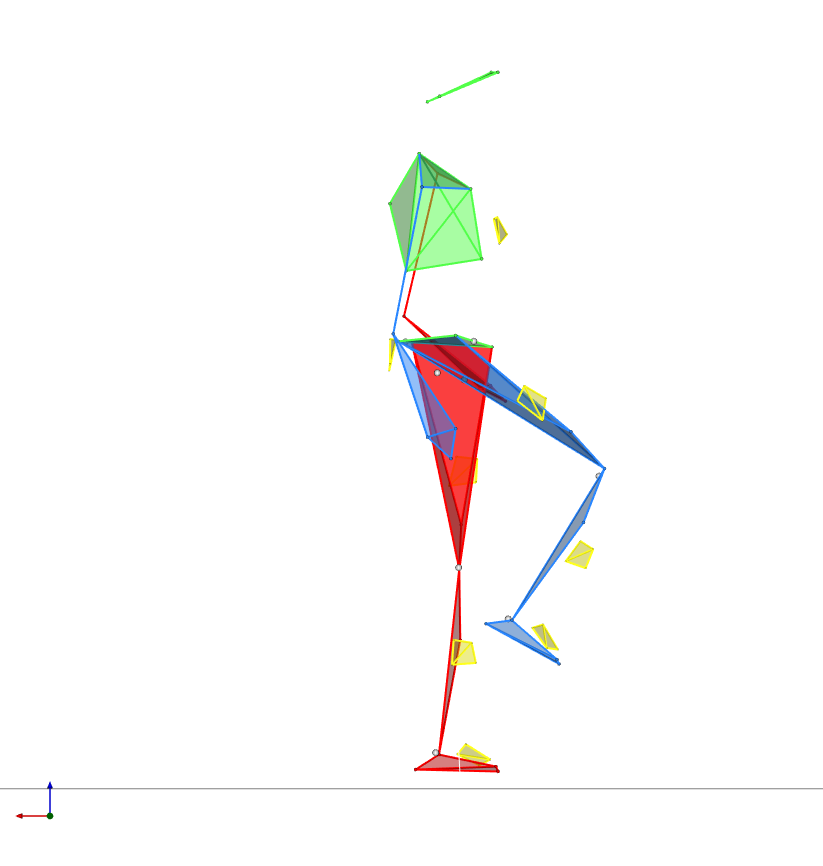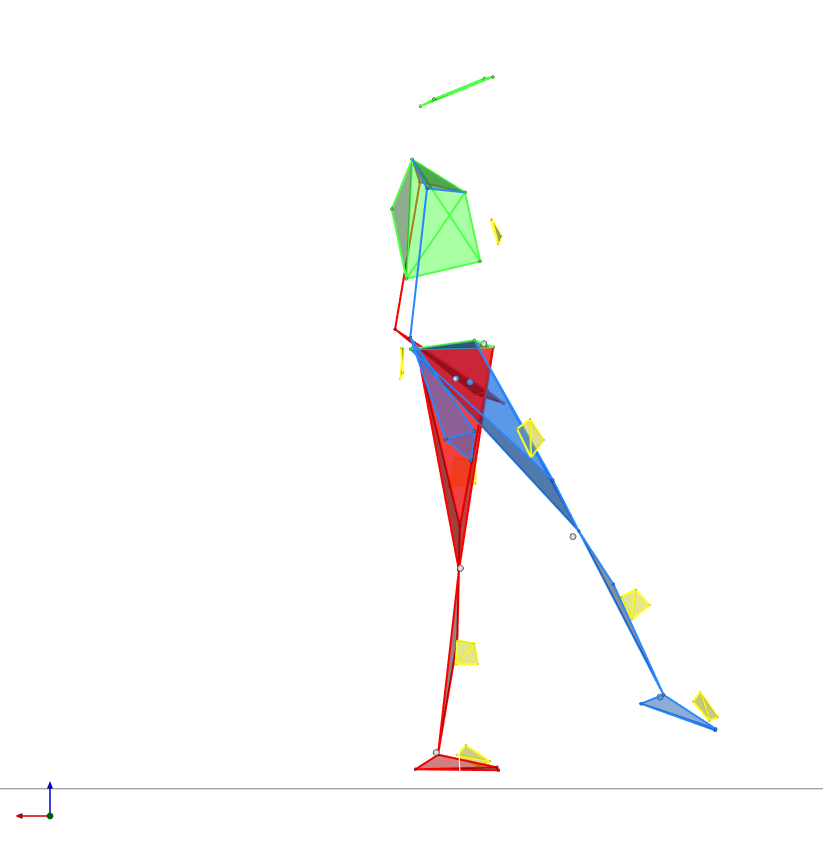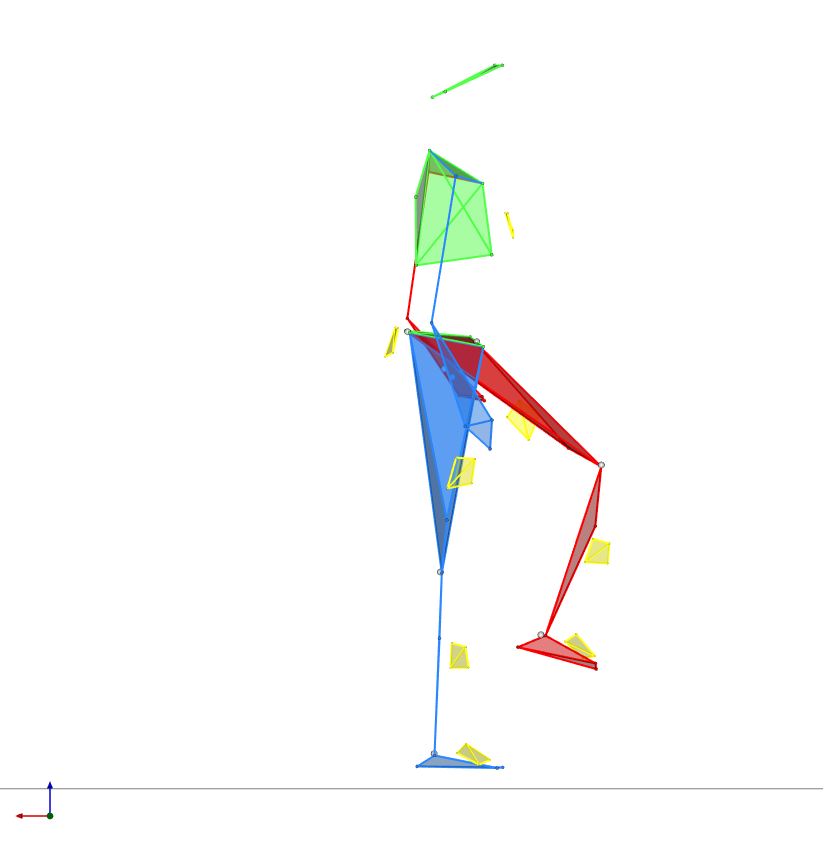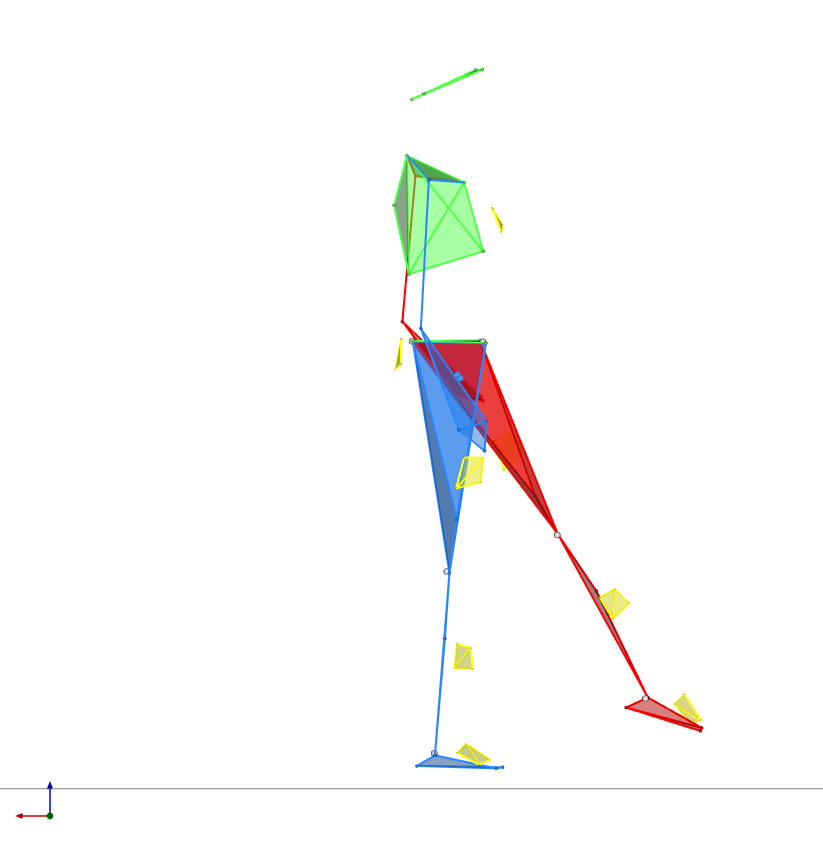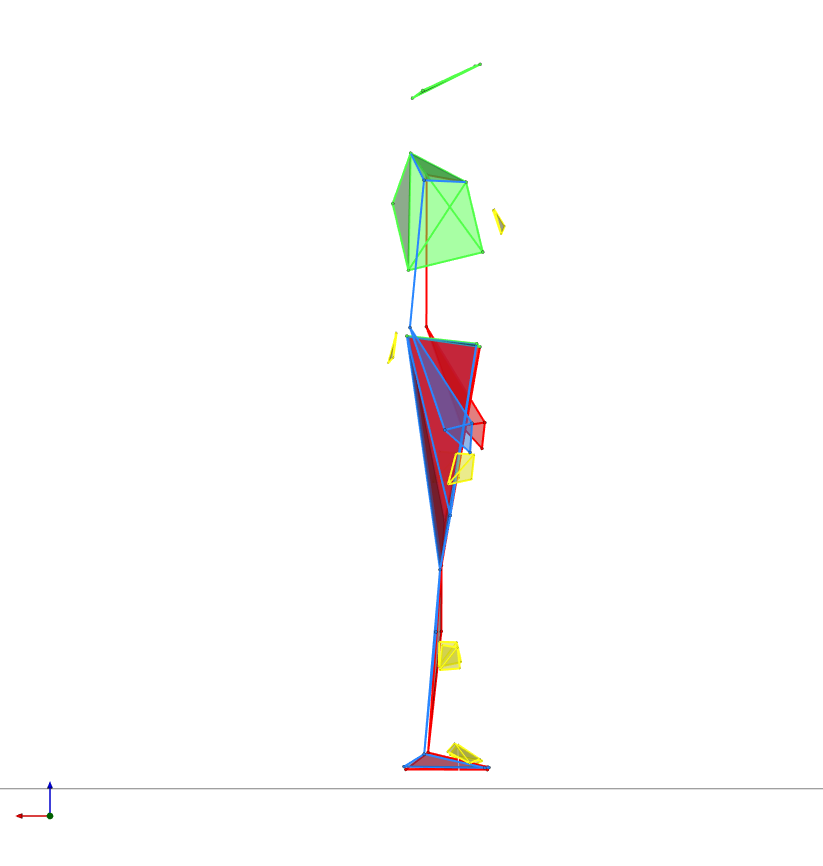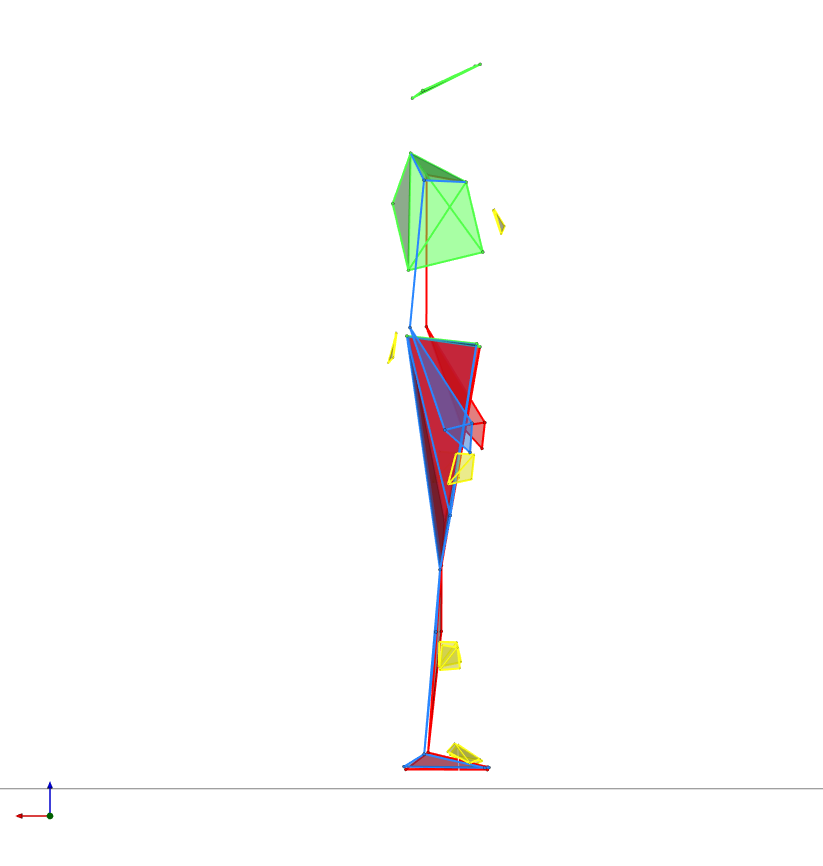 | - Hold on to a chair on your side so you don't lose your balance - Other arm at the side of the body - Movement aligned in sagittal plane - 5 repetitions per side | *Raise the knee as high as possible, then extend the leg and the foot, 5 repetitions on the right, then 5 repetitions on the left side* |
| **Hip ab/adduction** | 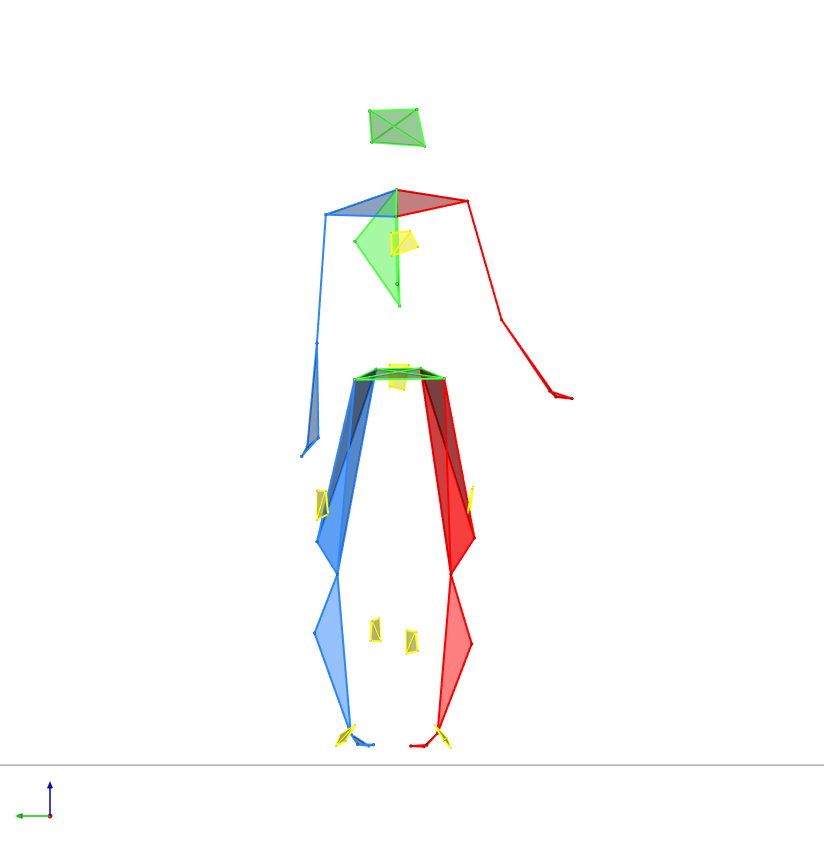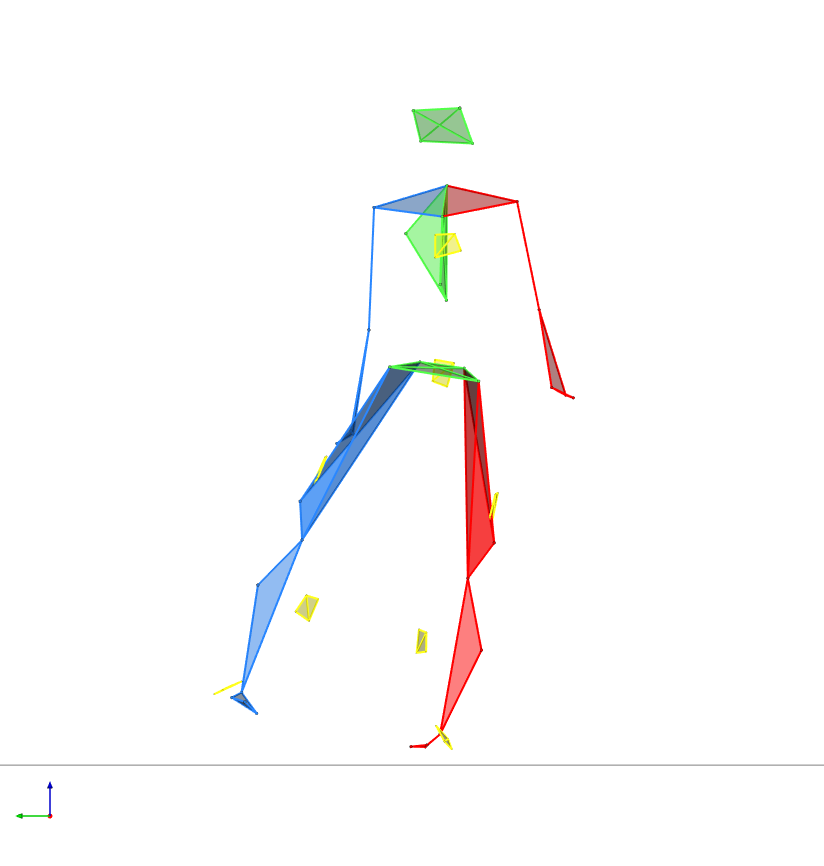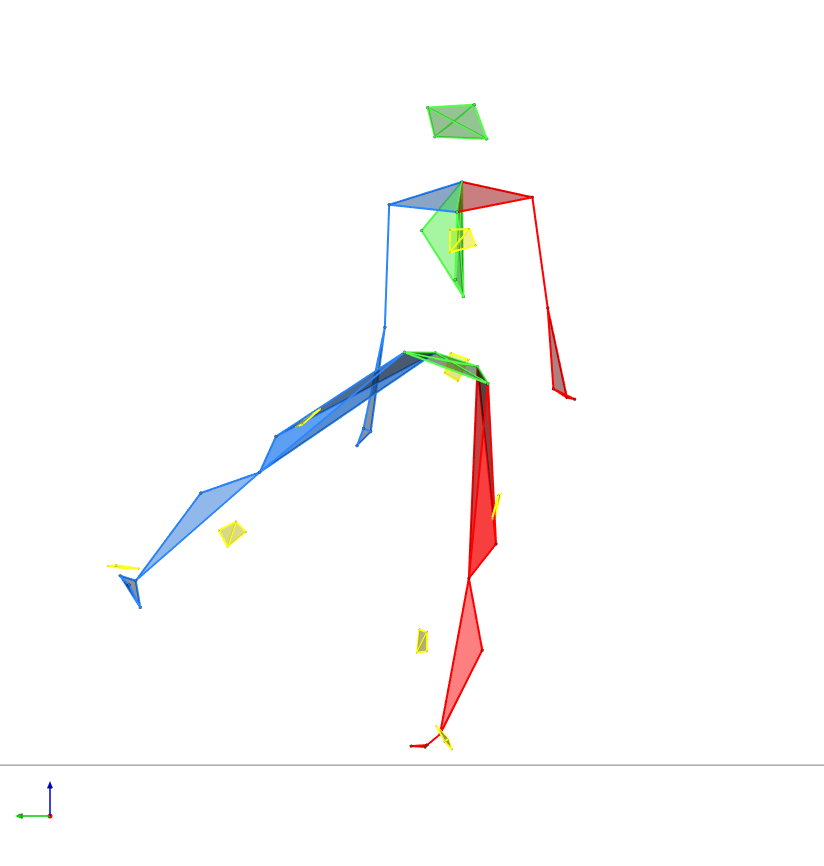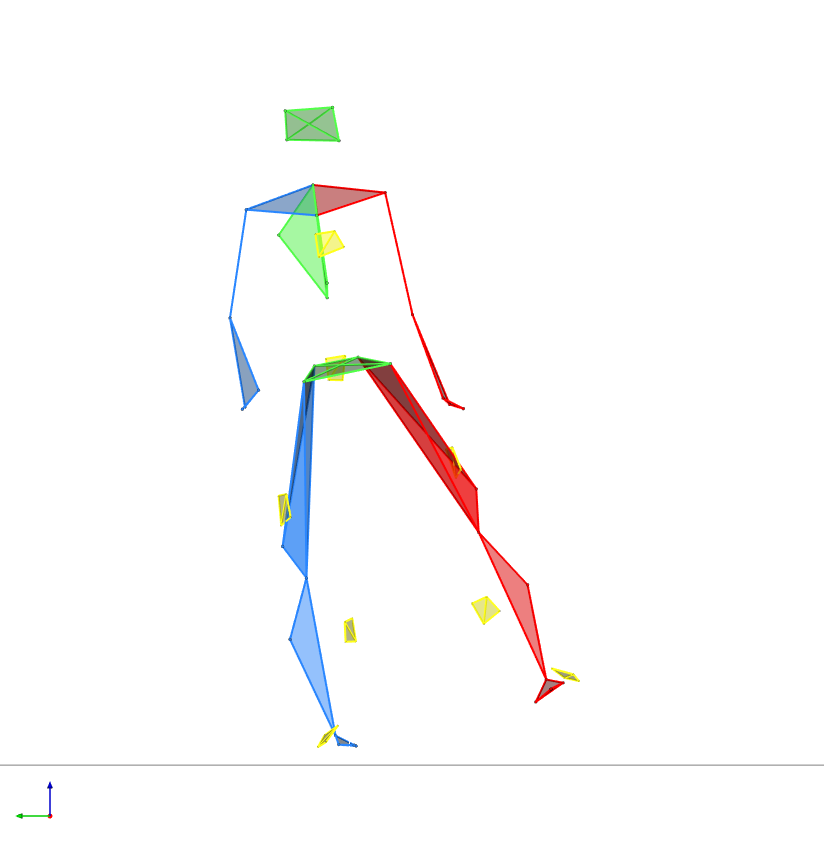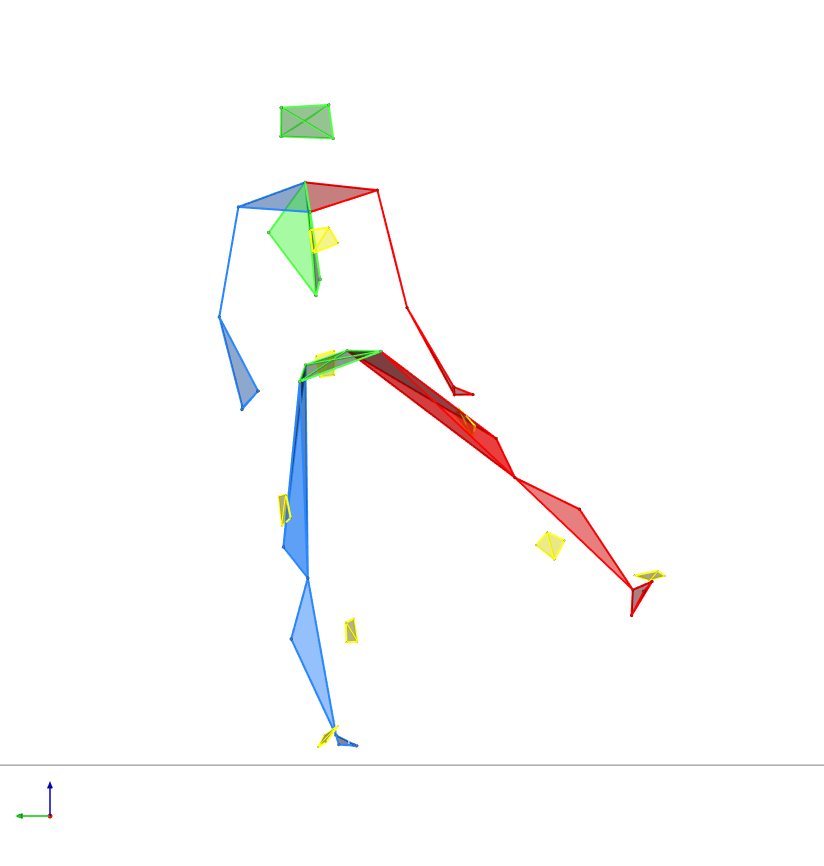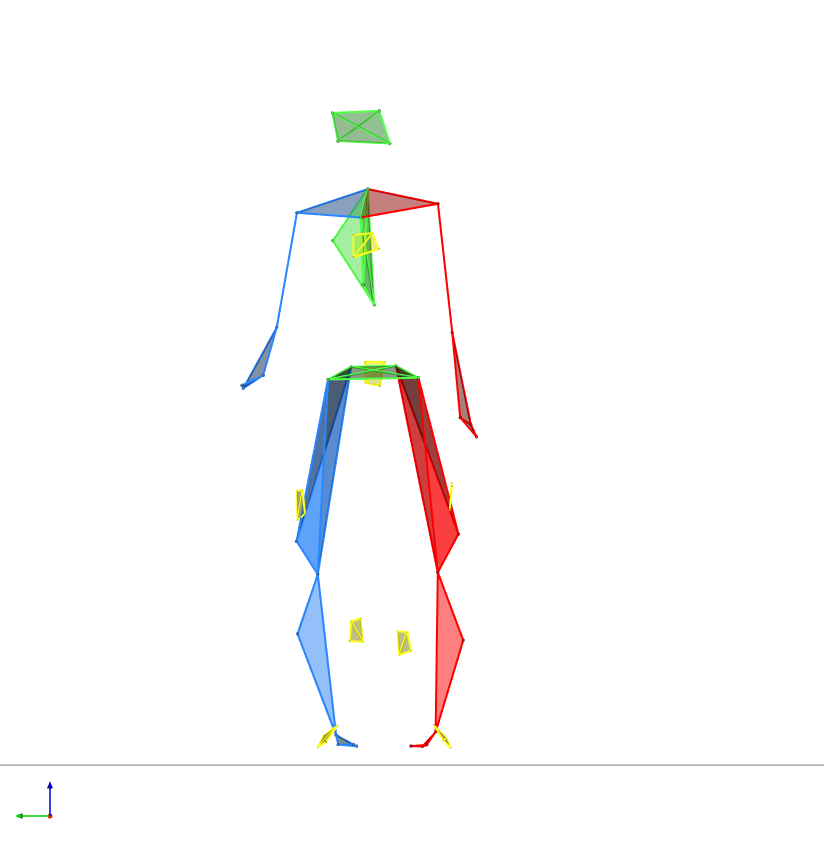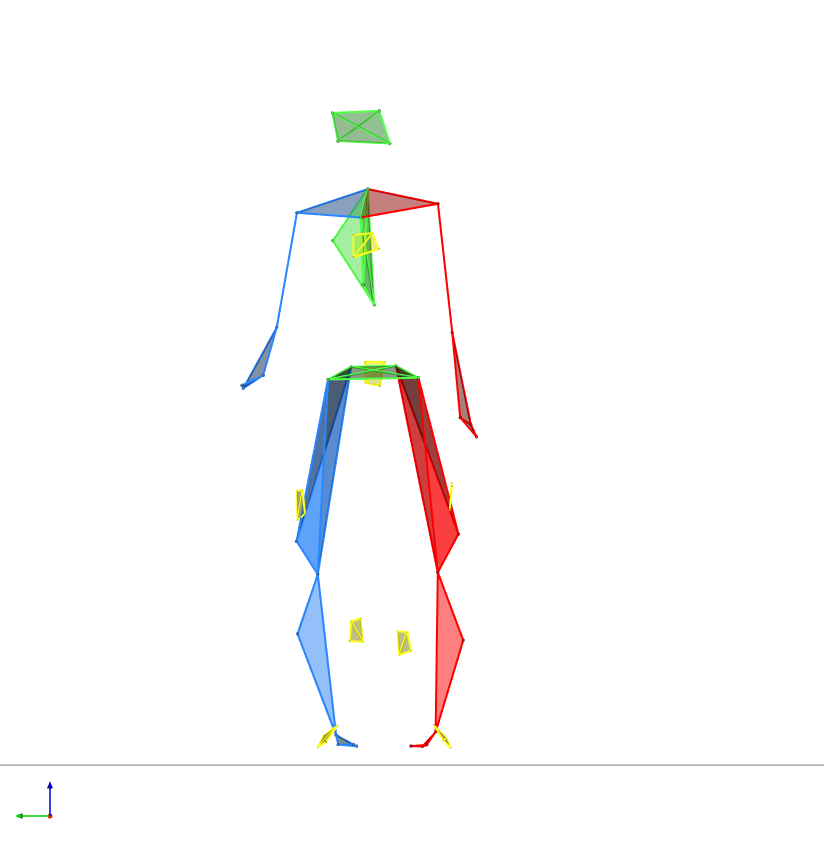 | - Hold on to a chair on your side so you don't lose your balance - Other arm at the side of the body - Movement aligned in coronal plane - 5 repetitions per side | *Lift the leg to the side and rest the leg, 5 repetitions on the right, then 5 repetitions on the left side* |
| **Hip rotation** | 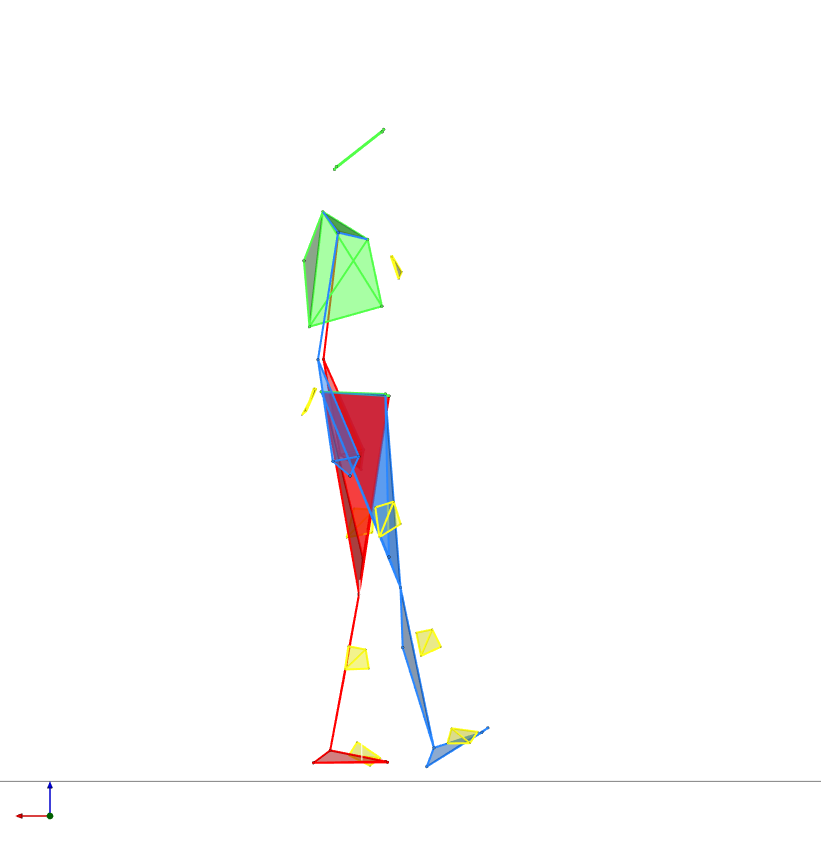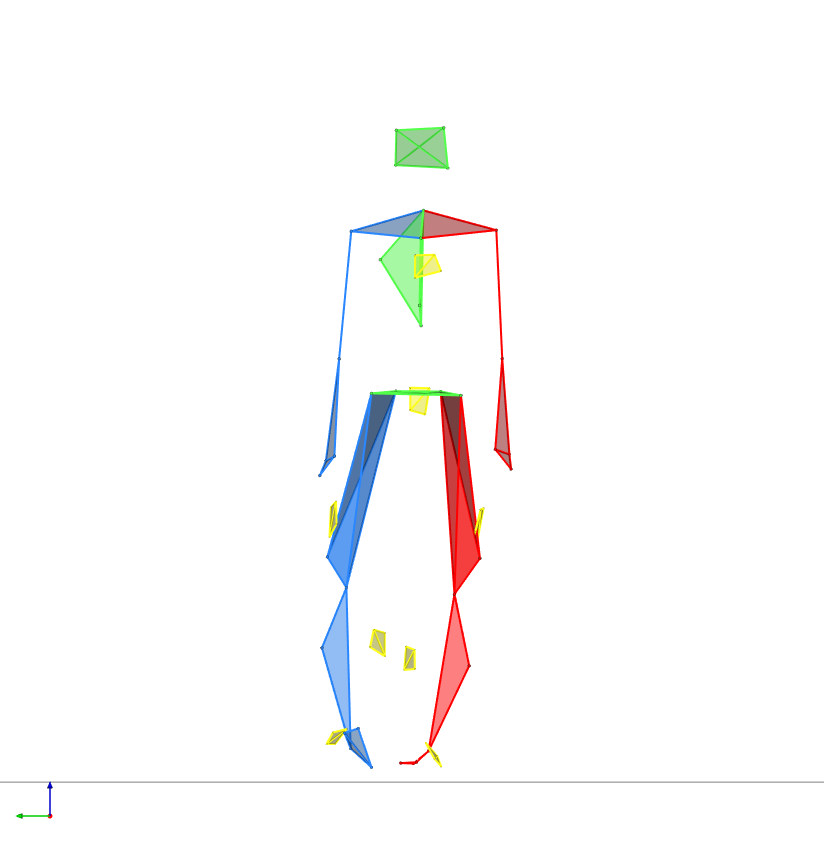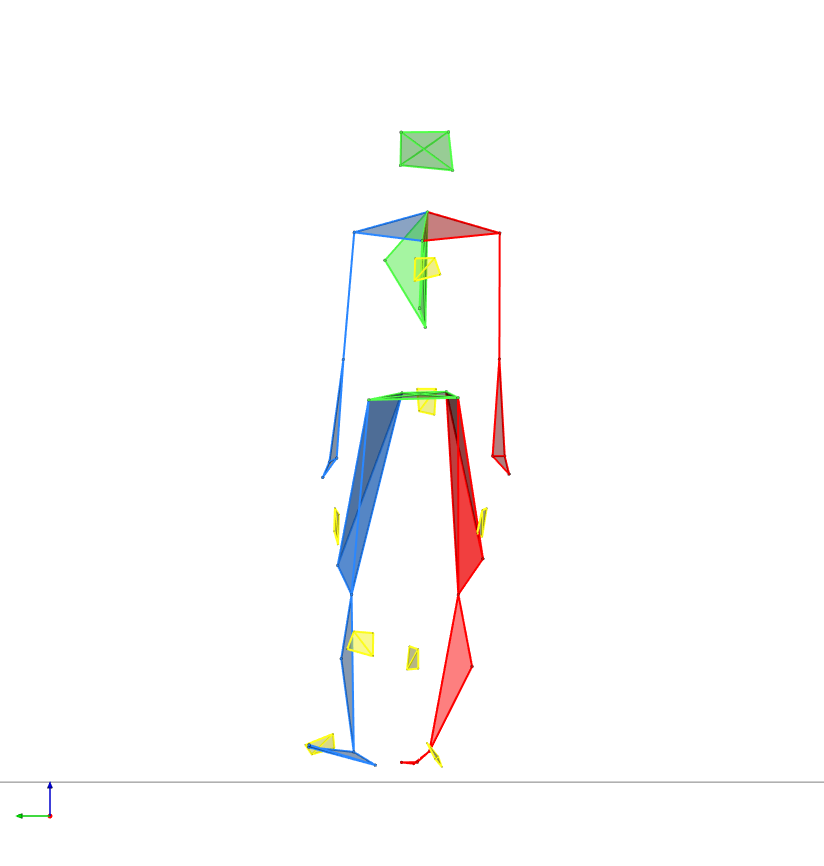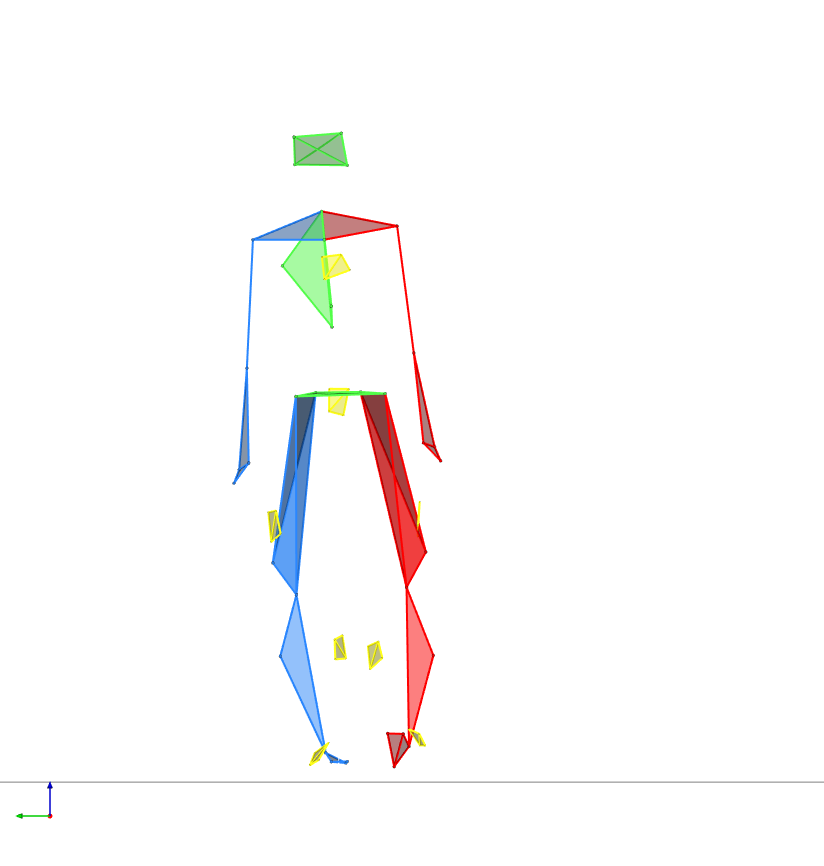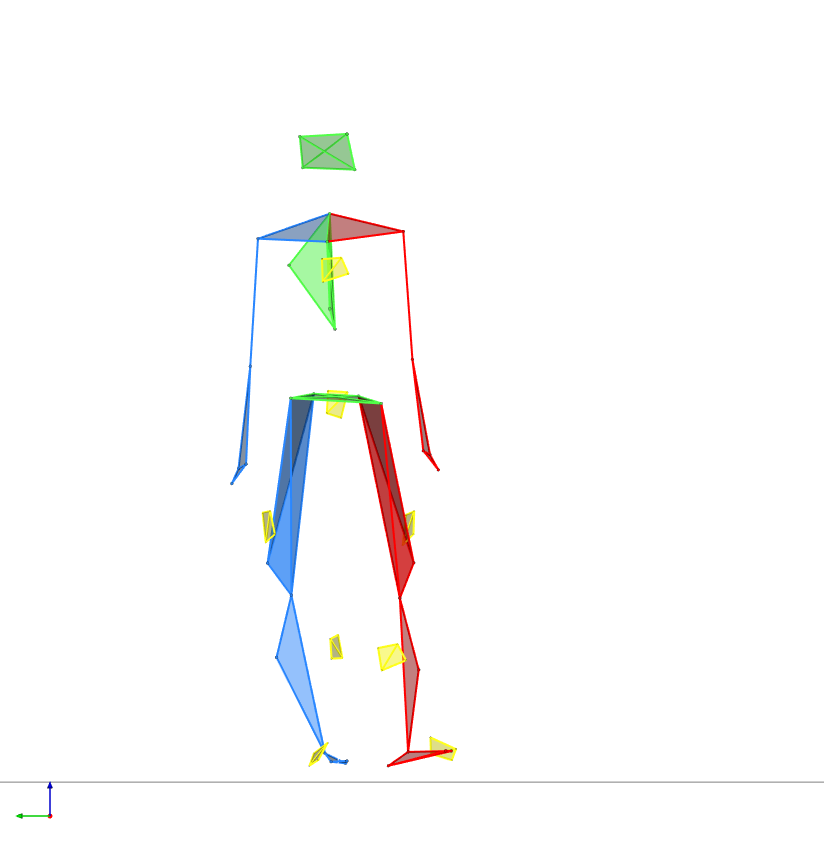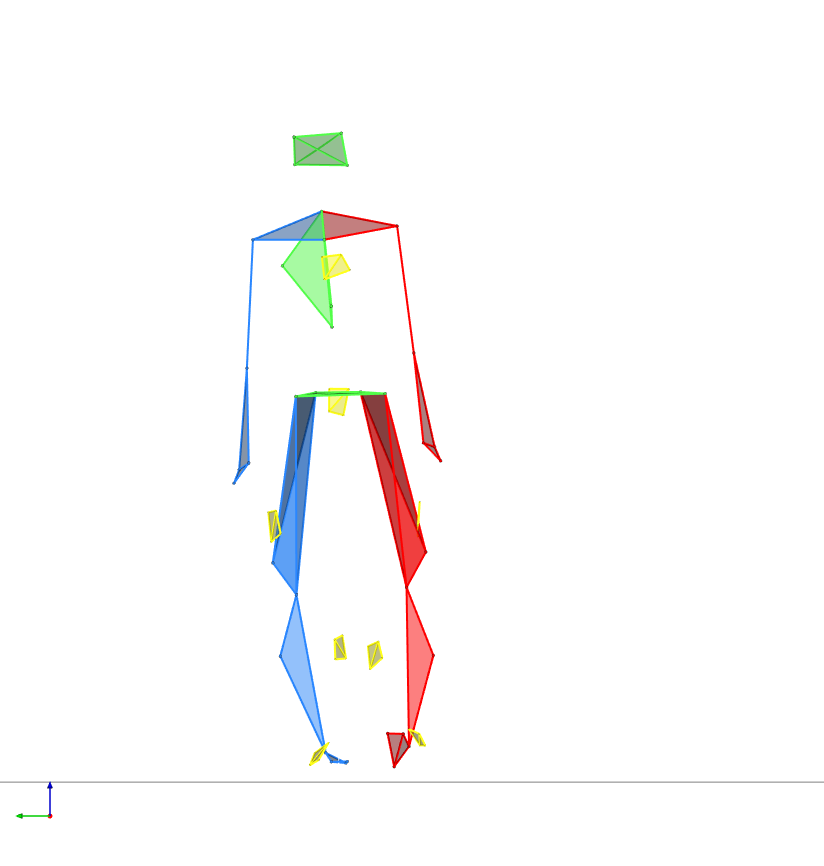 | - Foot about 20cm in front of the other - Legs extended - Heel on the ground - Rotations of the leg leads to hip rotations - 5 repetitions per side | *Extend the leg, place only the heel on the ground slightly forward and rotate the leg, the heel must remain in contact with the ground, 5 repetitions per side* |
| **Pelvis rotation** | 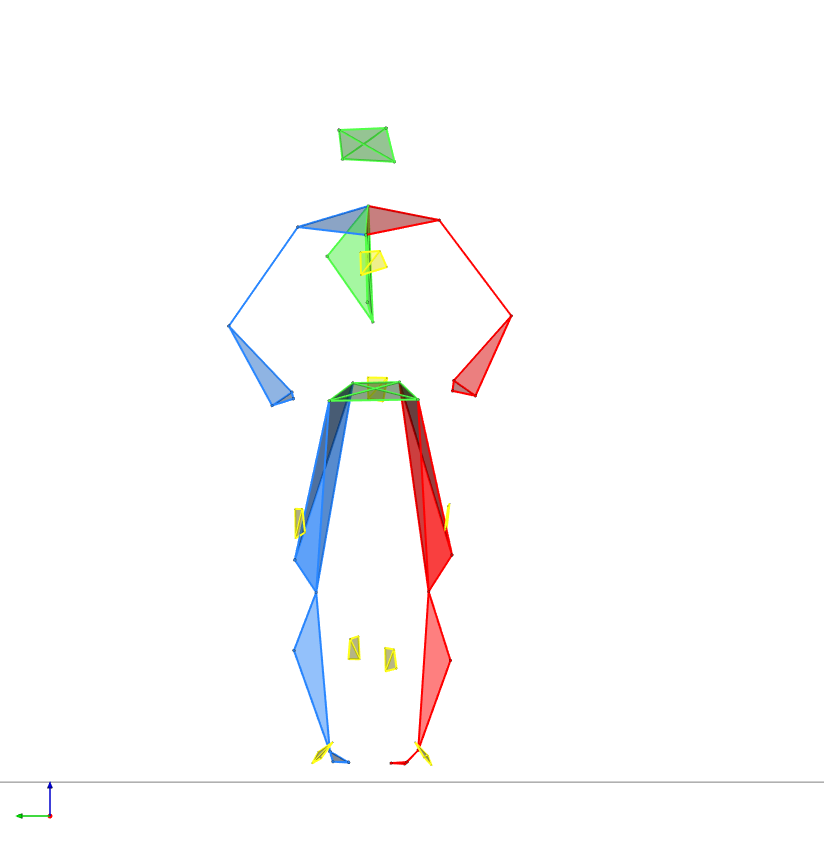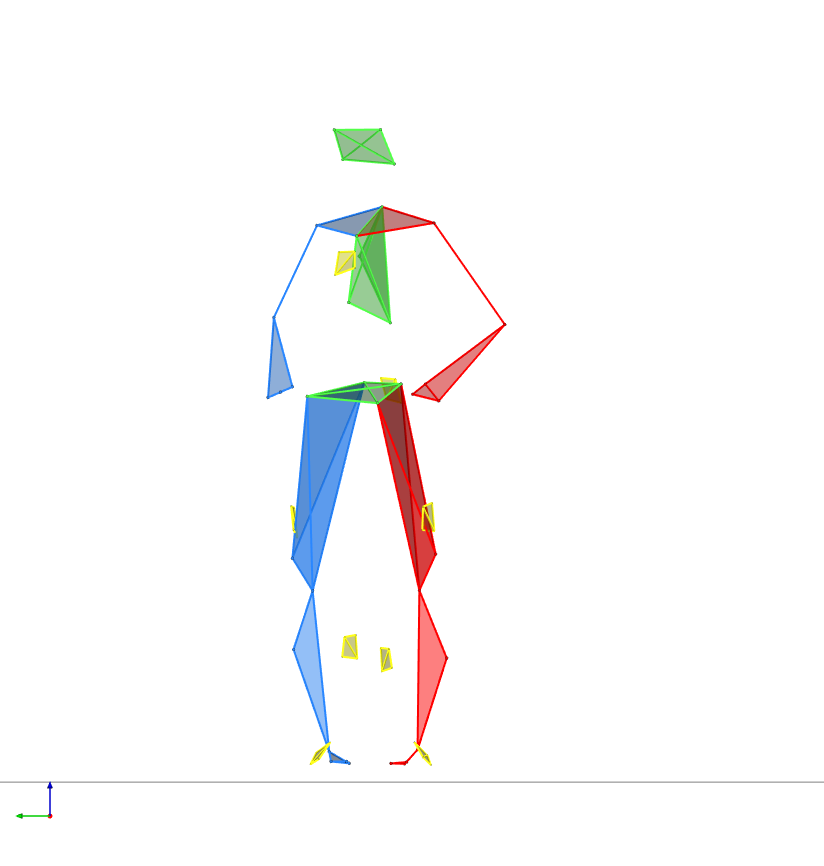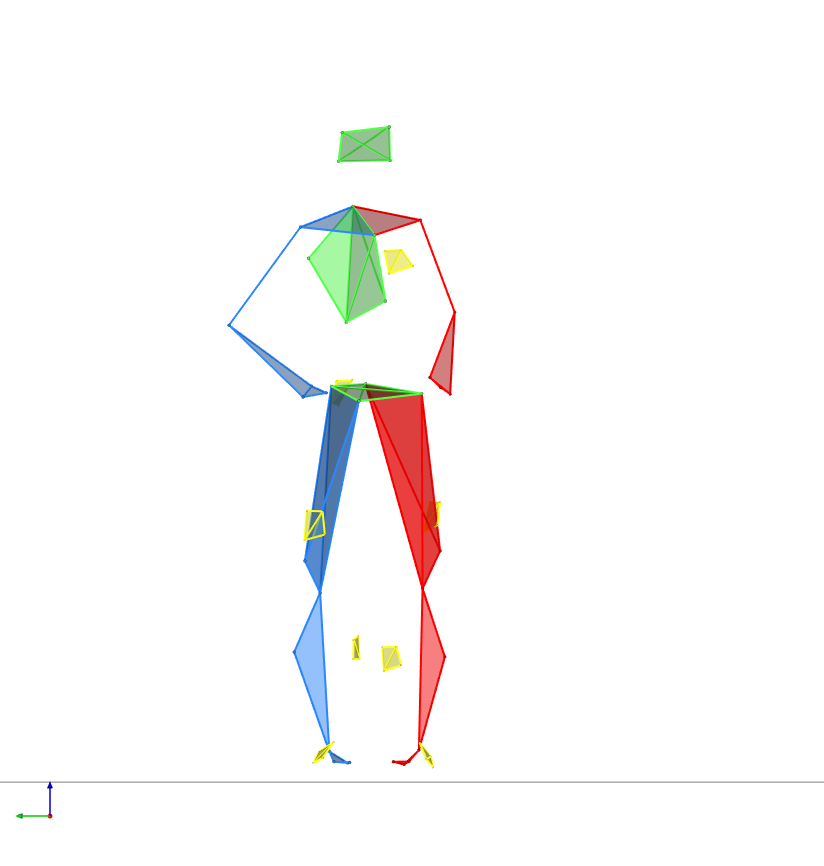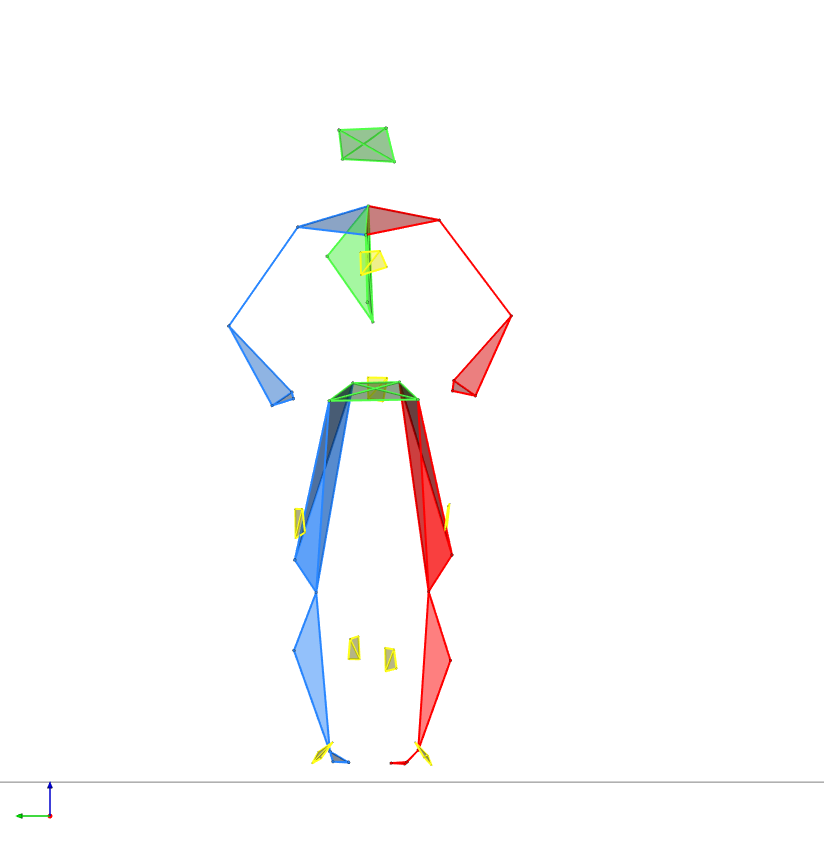 | - Hands on hips - Legs extended - Feet parallels - Only pelvis rotations - Legs should not move - Shoulders in line with the trunk - 5 repetitions | *Put your hands on your hips and rotate your pelvis without moving your legs, do not make 'circles' with your pelvis, 5 repetitions* |
| **Pelvis ante/retro version** | 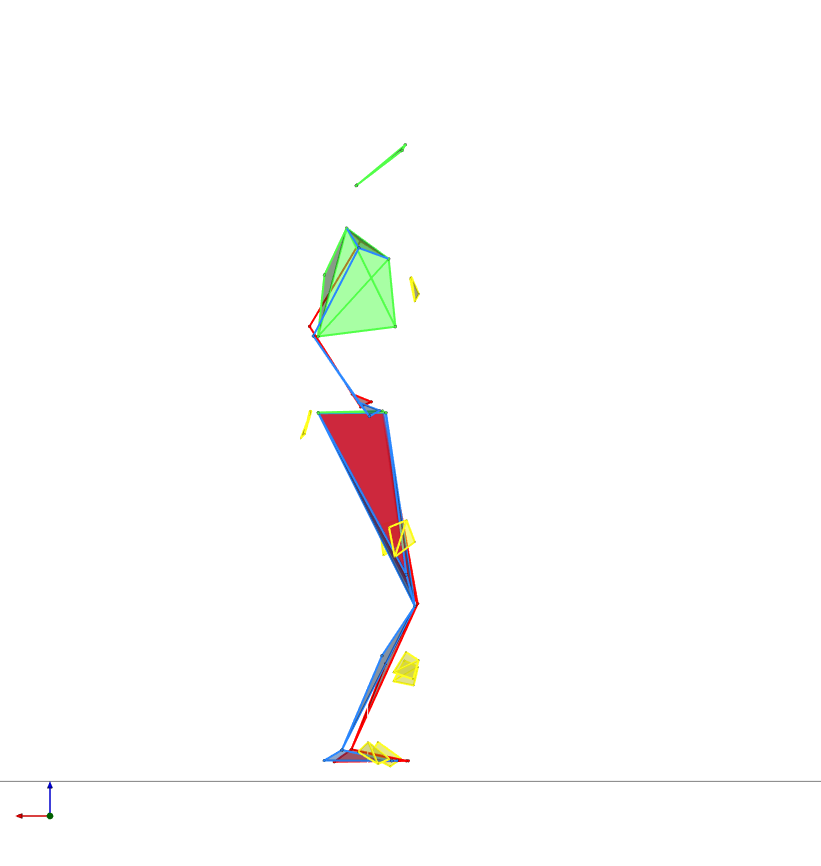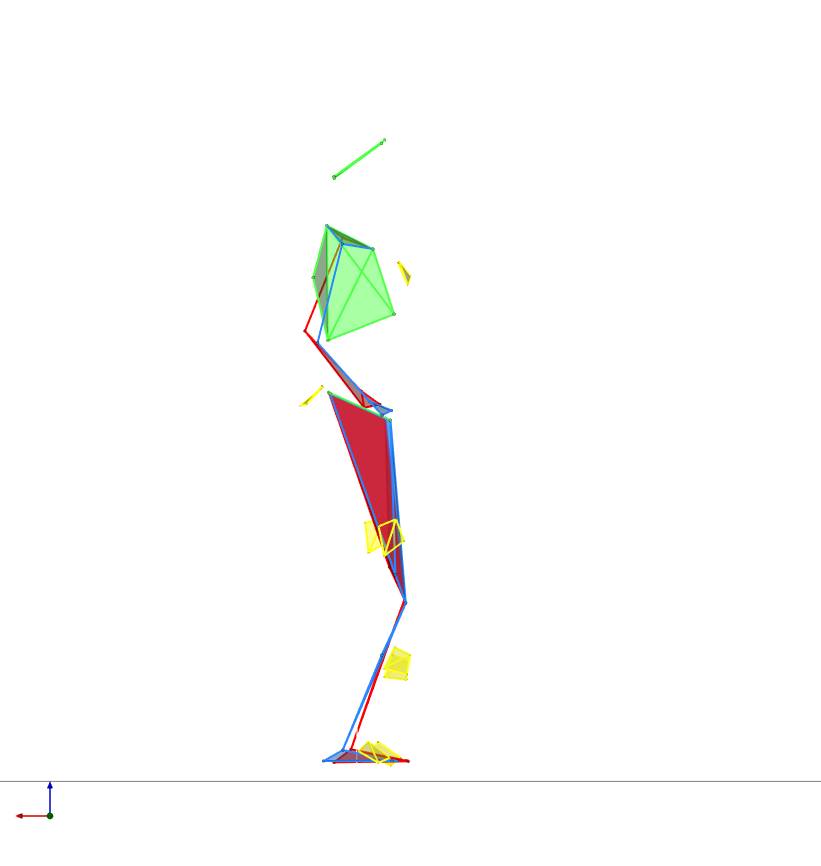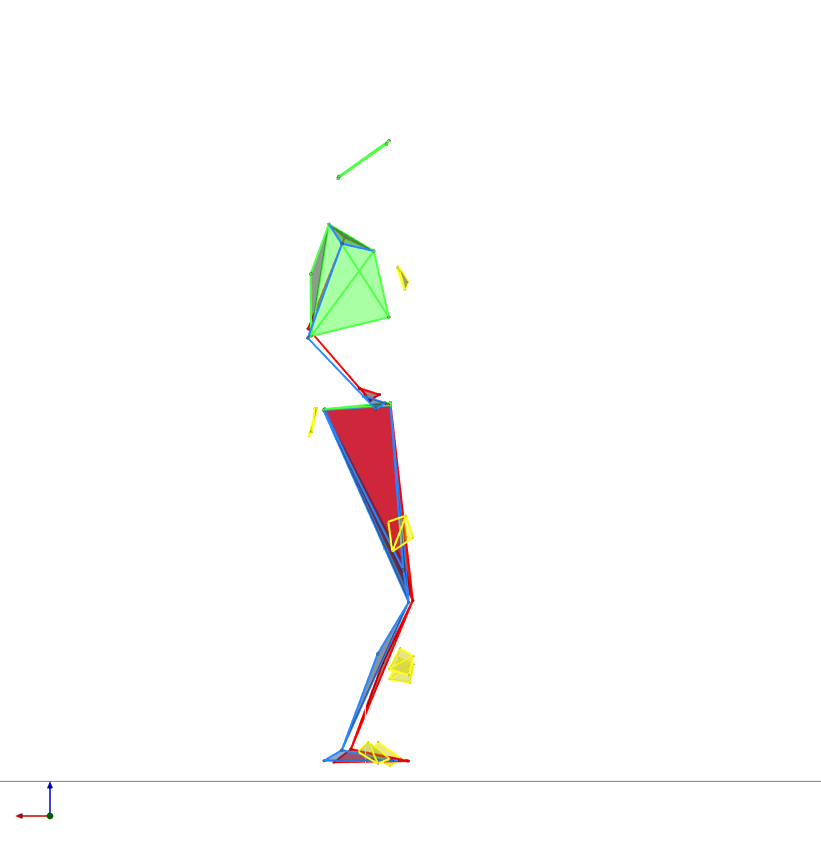 | - Hands on hips - Legs slightly bent - Feet parallels - Back straight - Only pelvis ante/retroversion - 5 repetitions | *Perform forward and backward movements of the pelvis while keeping the back straight and without moving the legs, 5 repetitions* |
| **Pelvis obliquity** | 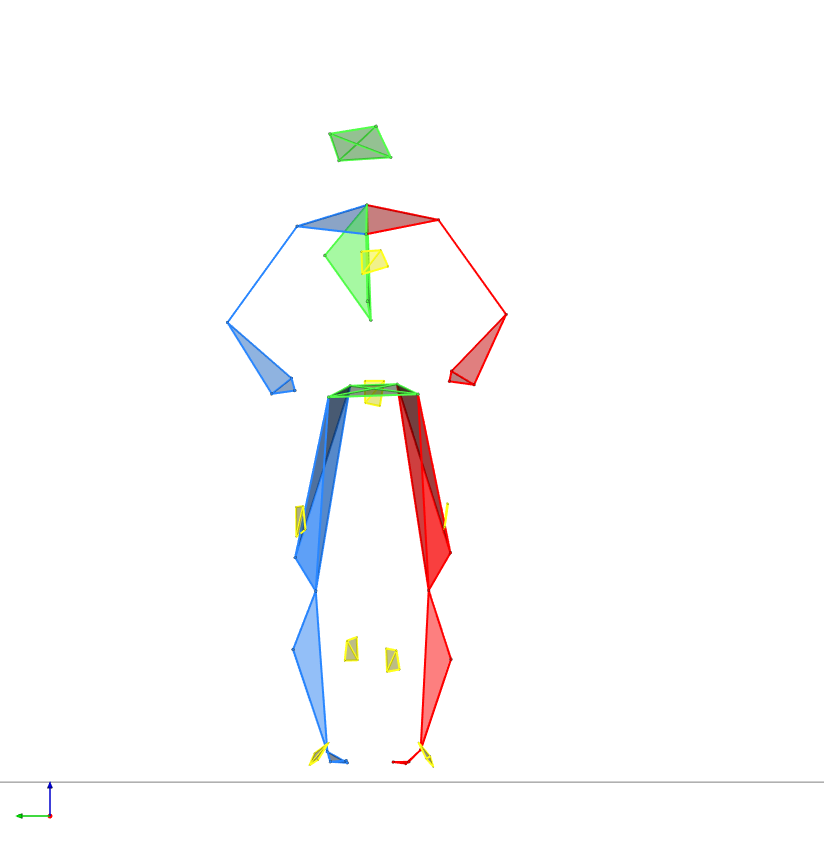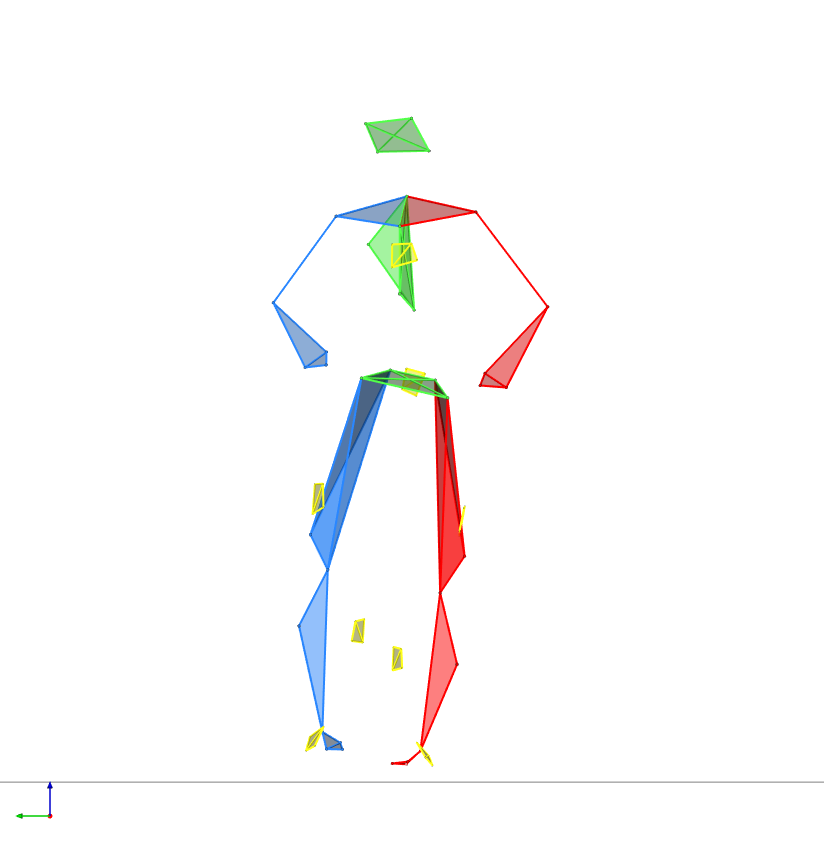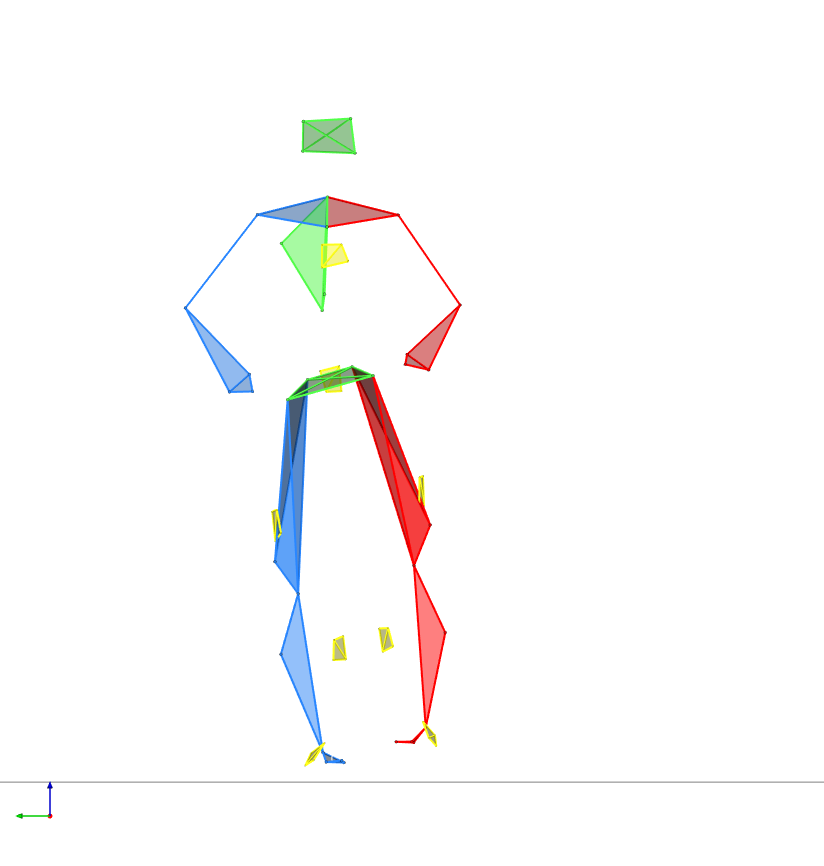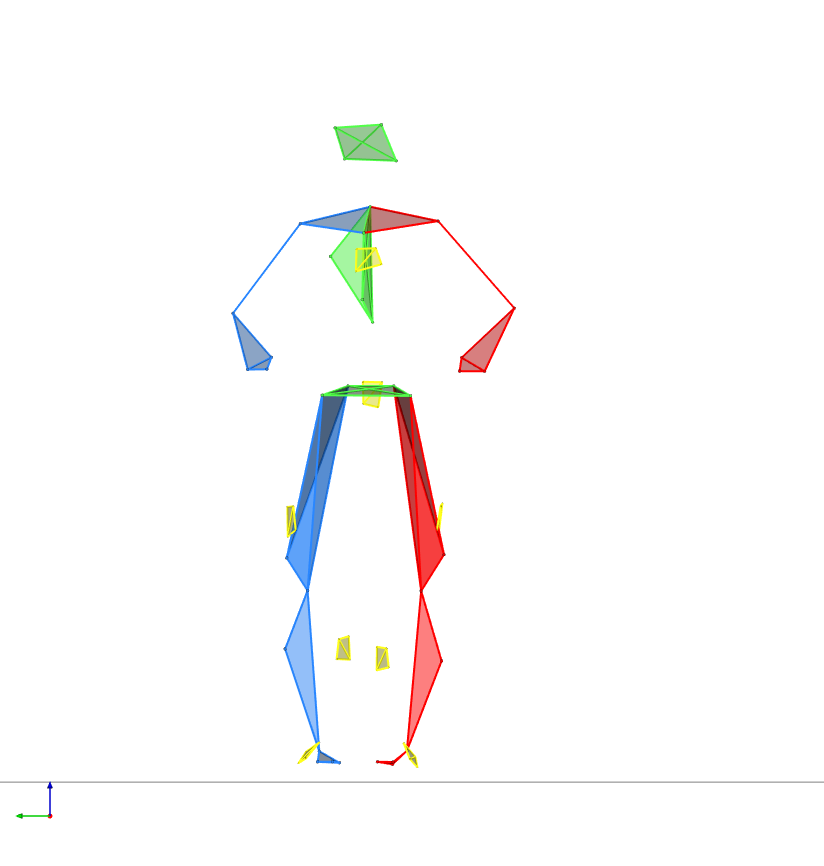 | - Hands on hips - Legs extended - Don’t move the leg that remains on the ground - Feet parallels - Back straight - 5 repetitions per side | *Perform up and down movements of the pelvis while keeping the back straight and without moving the leg that remains on the ground, 5 repetitions per side* |
| **Gait (self-selected, slow, fast)** | 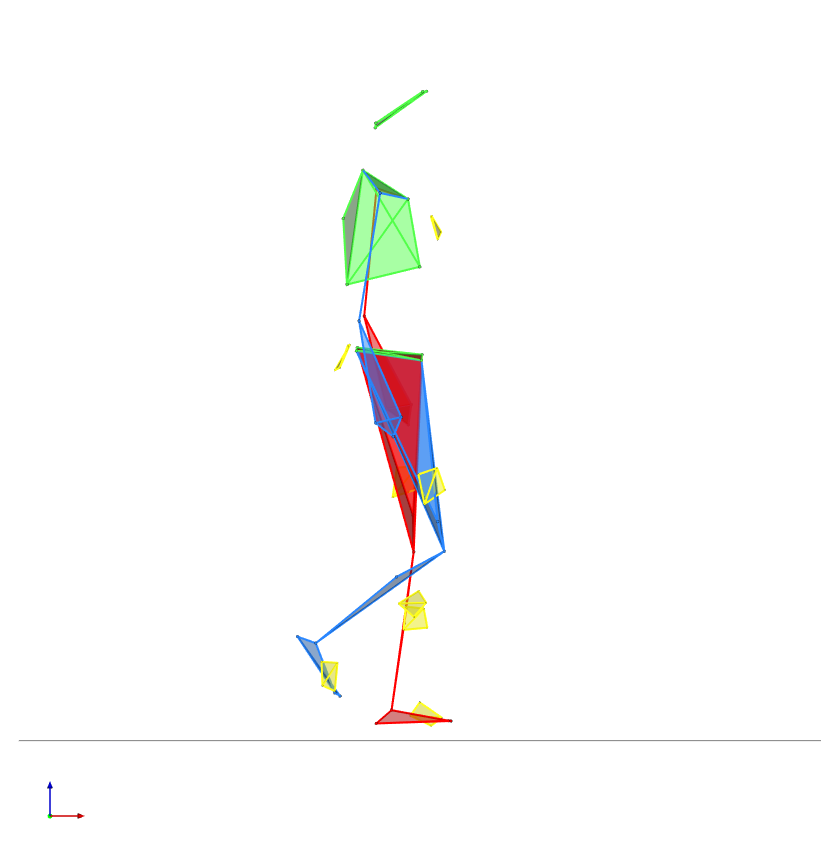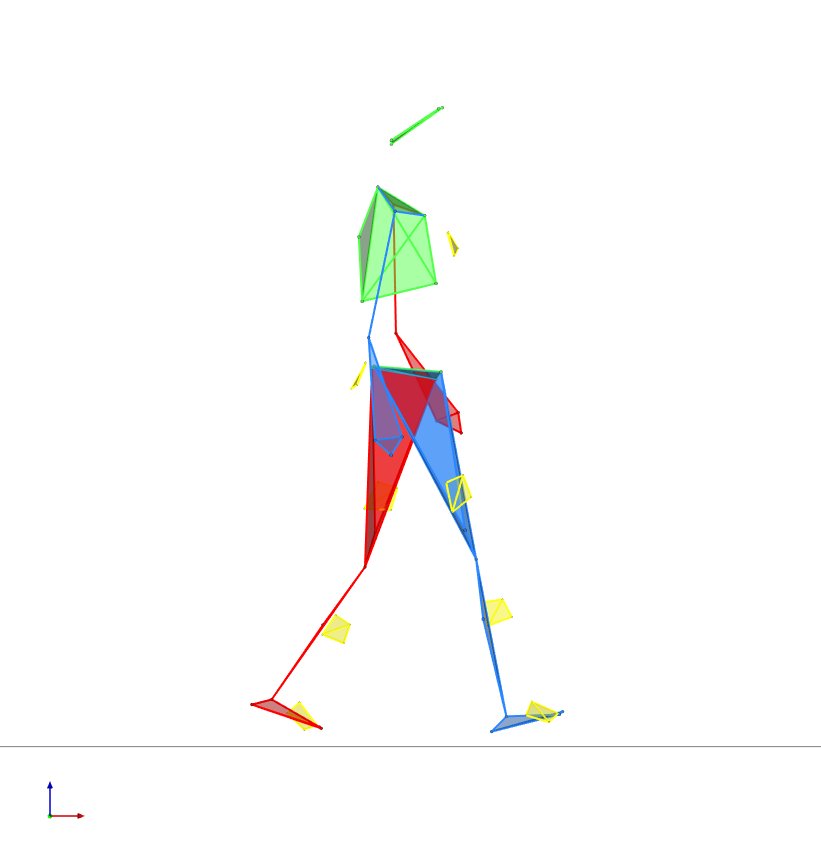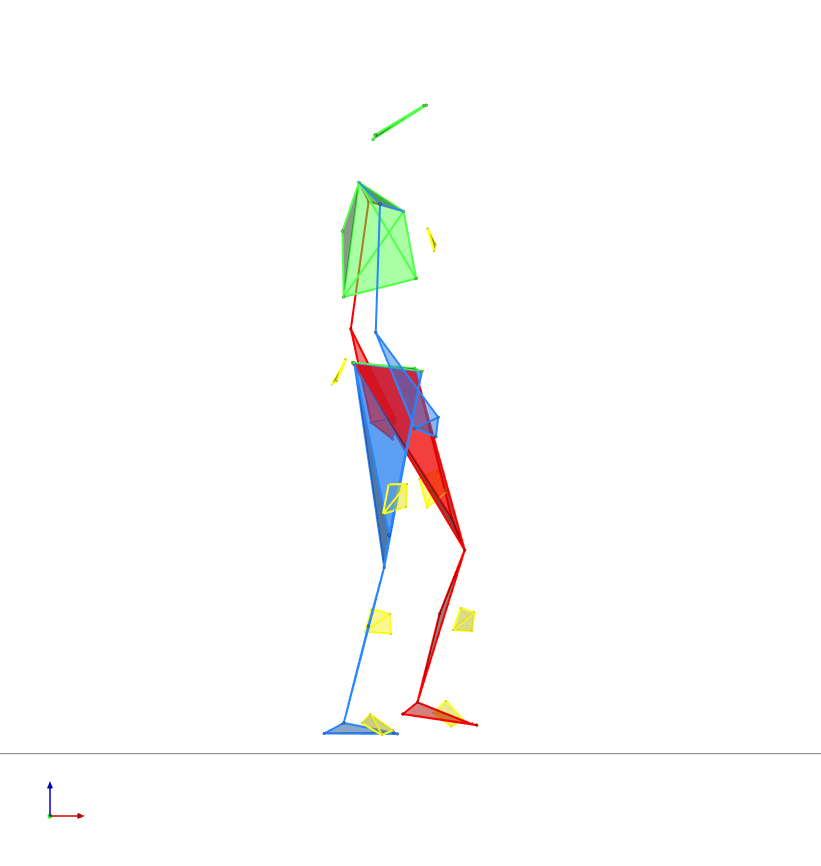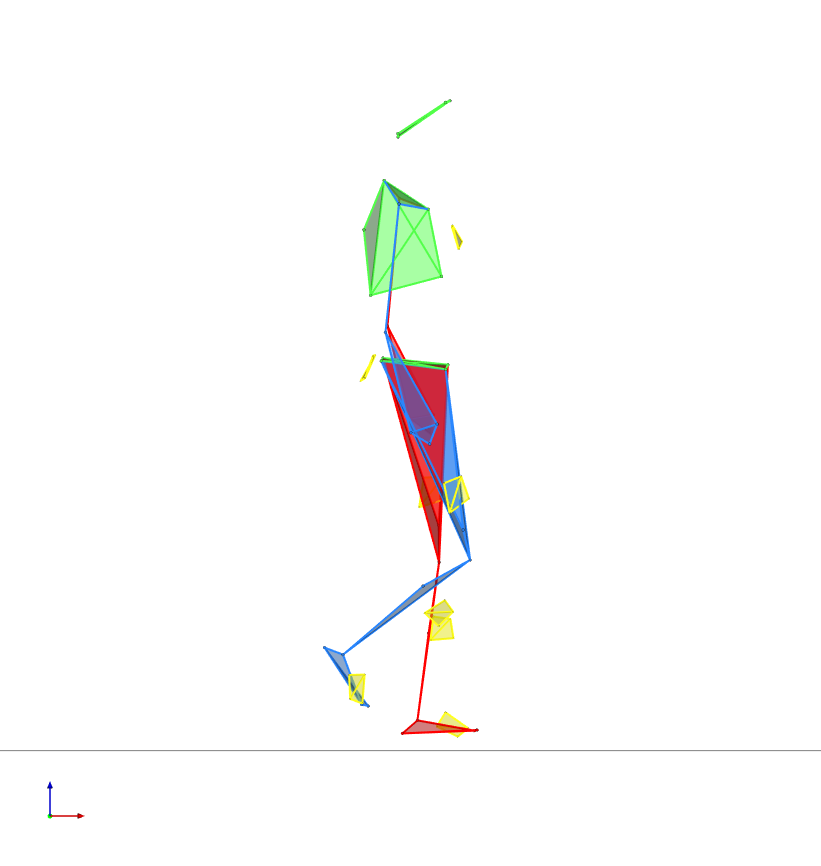 | - Self-selected speed | *Walk naturally to the other end of the walkway at a comfortable speed* |
|  |  | - Slow speed | *Walk more slowly than before to the other end of the walkway* |
|  |  | - Fast speed | *Walk quickly to the other end of the walkway but do not run* |
| **Running** | 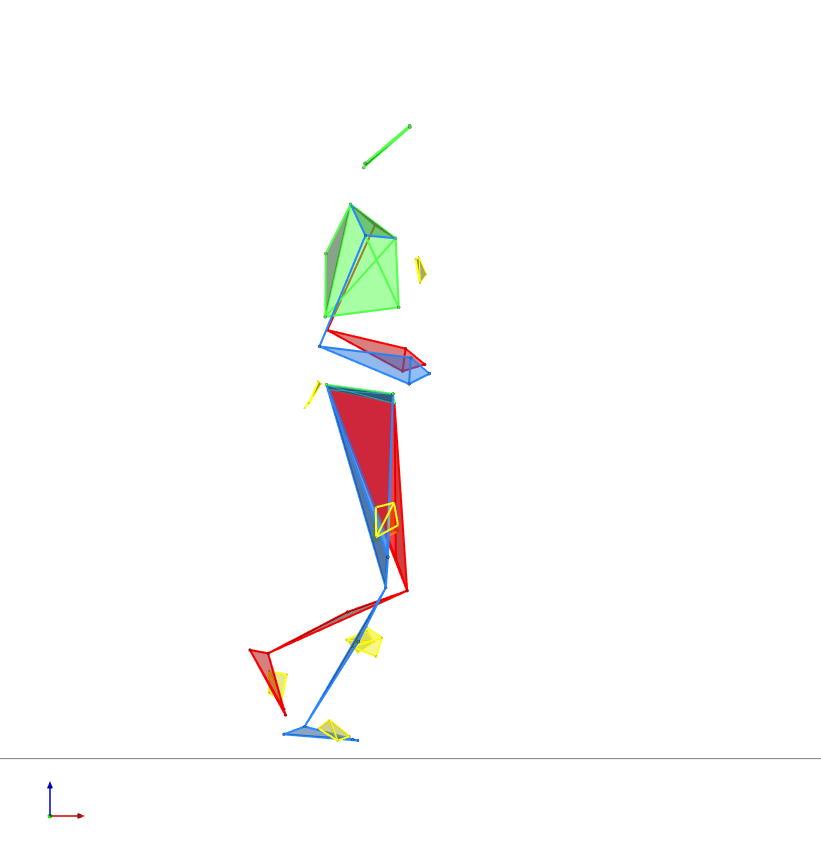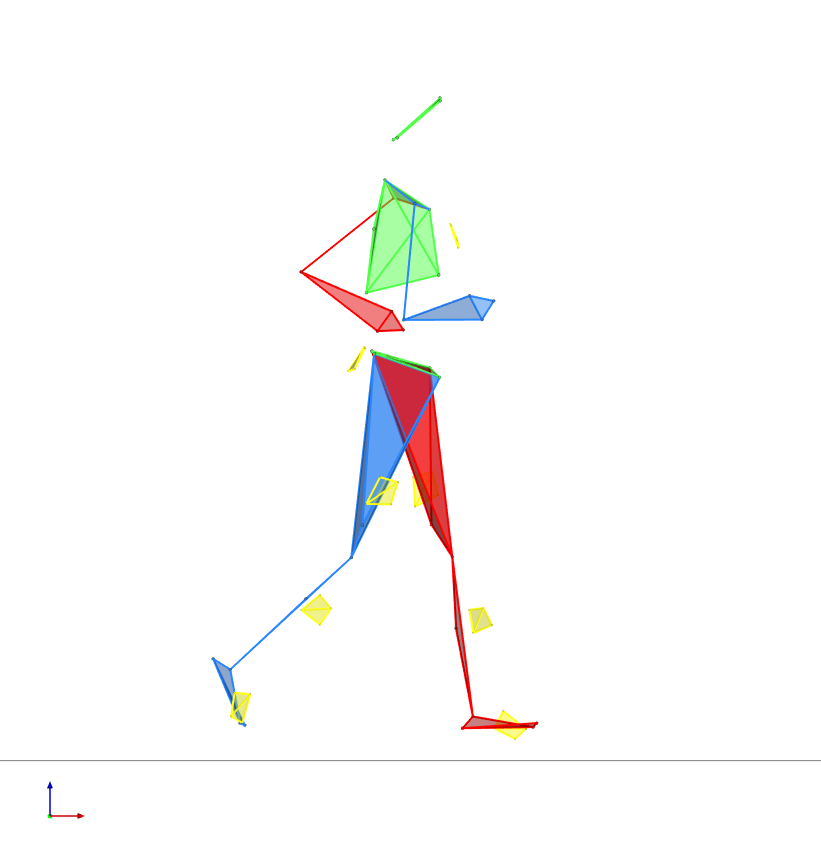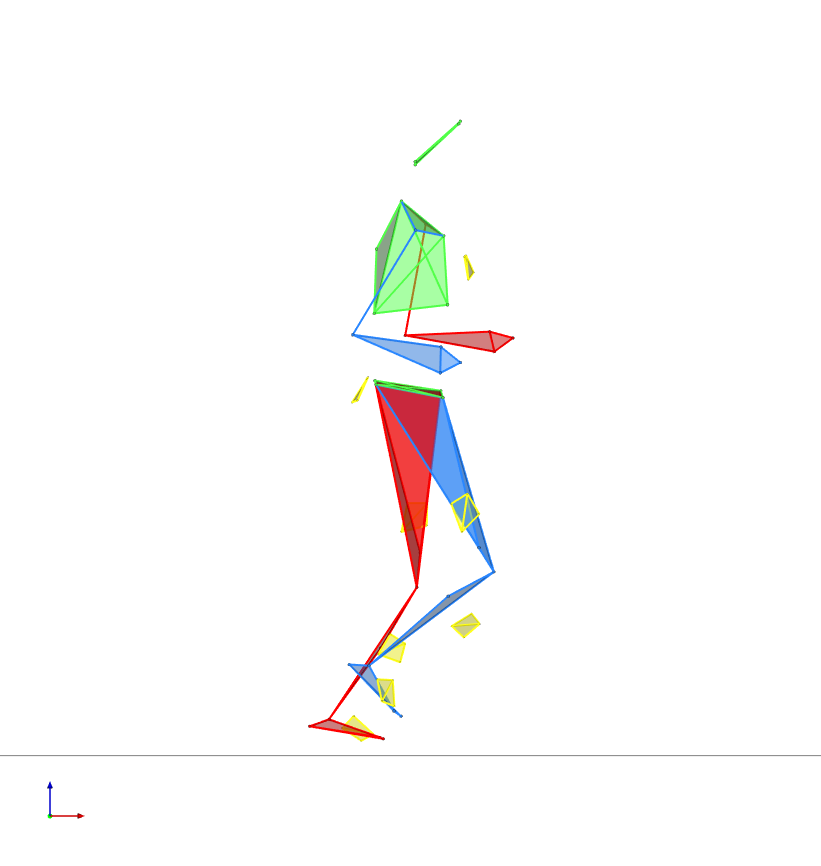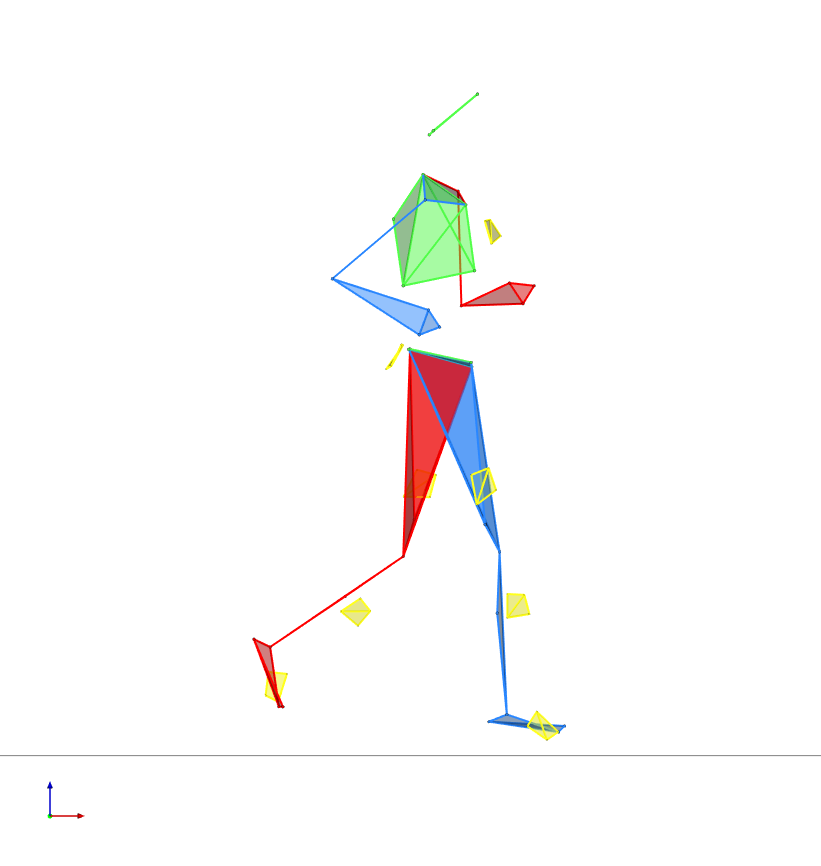 | - Run - No sprint | *Run to the other end of the walkway without sprinting* |
| **Sit to stand** | 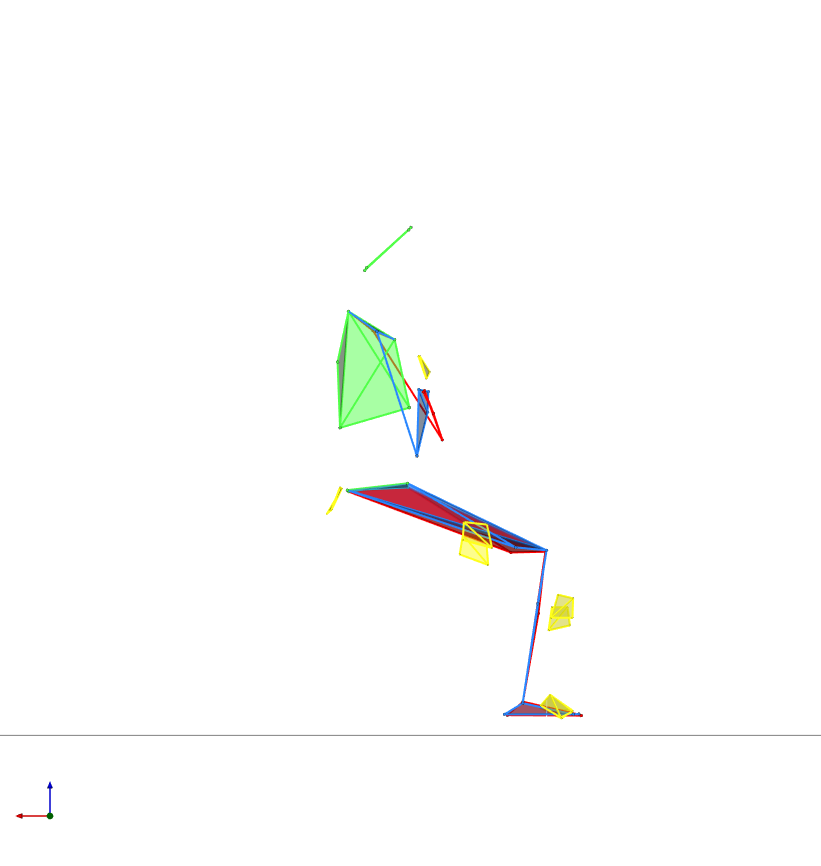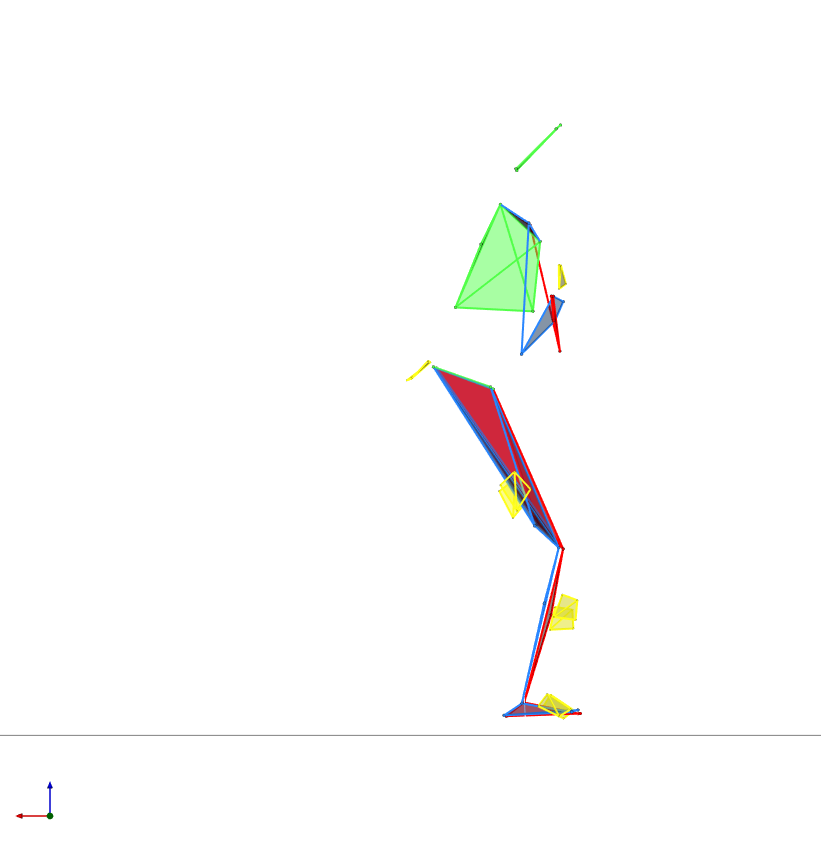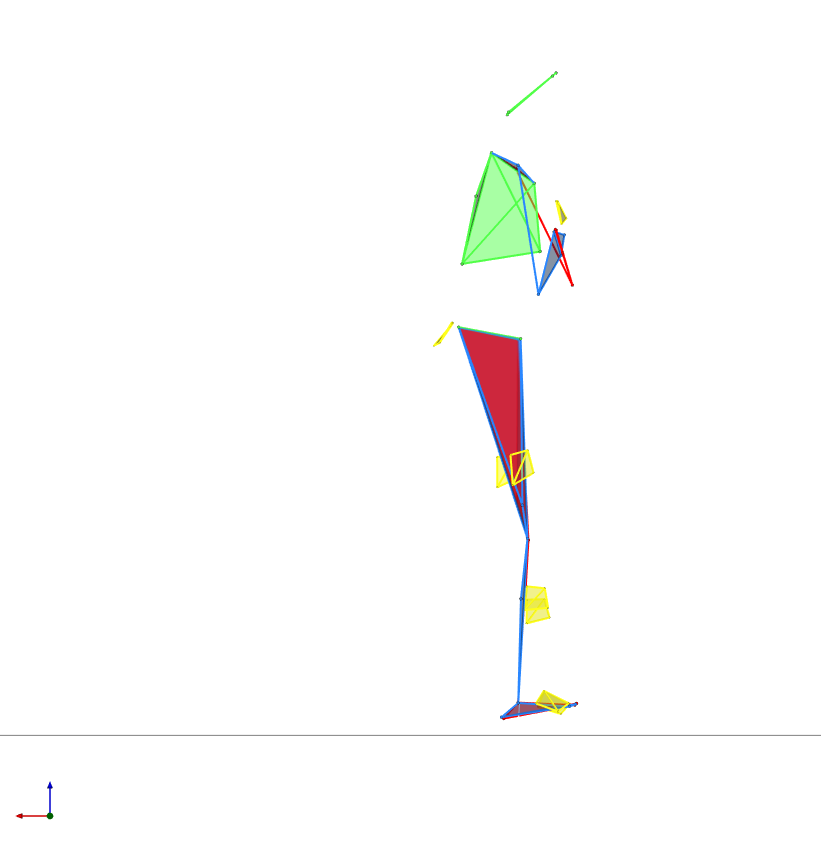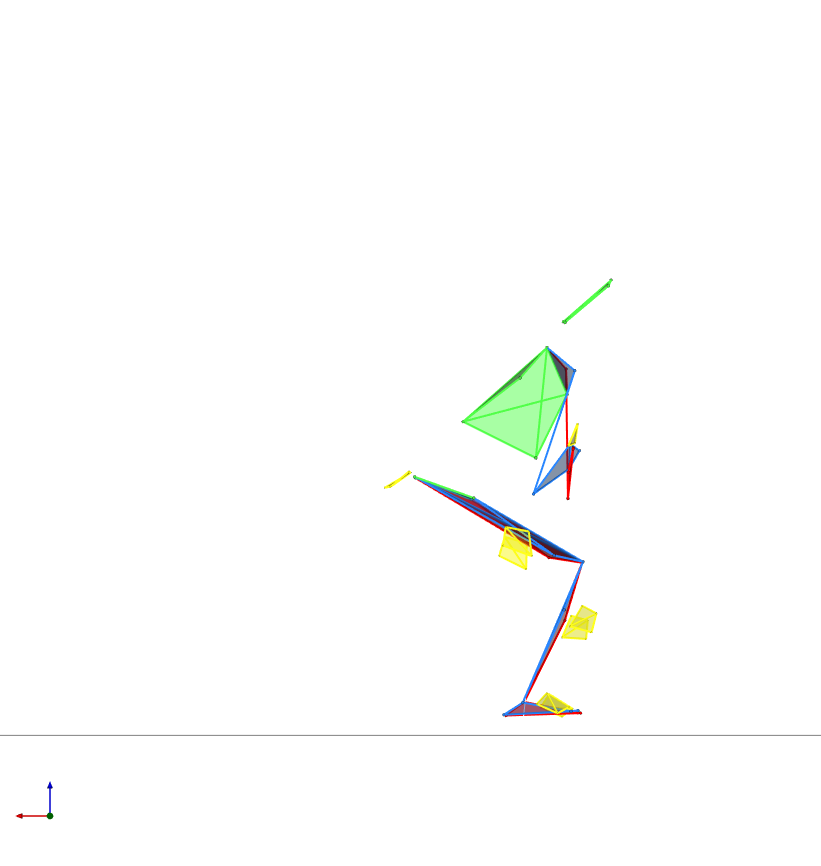 | - Hands on stomach/torso to avoid marker occlusion - 5 repetitions | *Stand up and sit down 5 times as fast as possible* |
| **Timed Up and Go** | 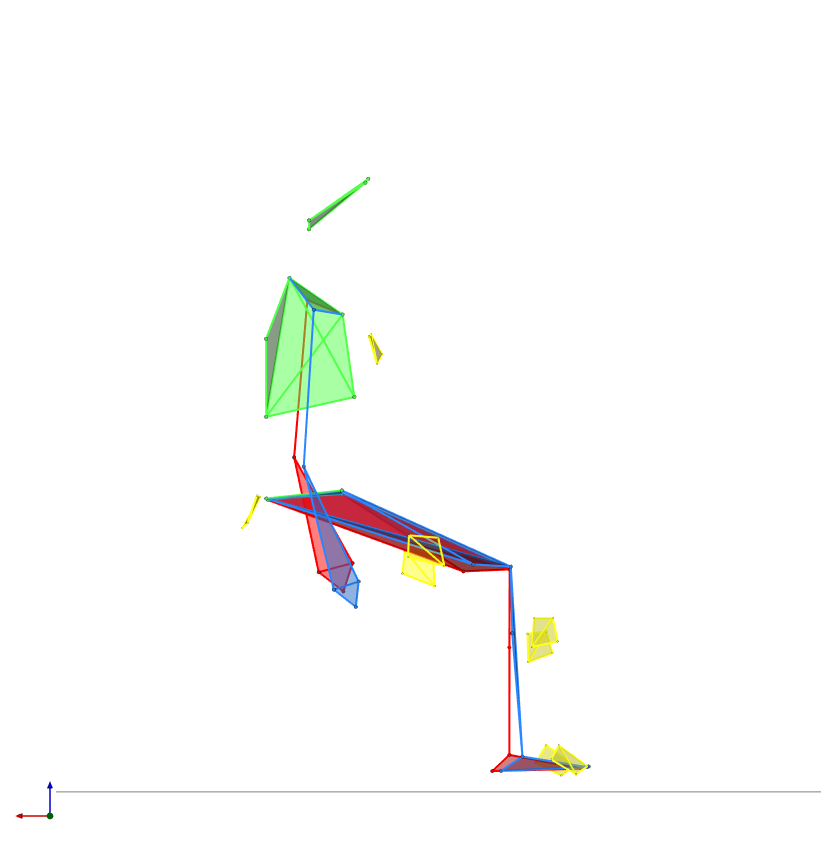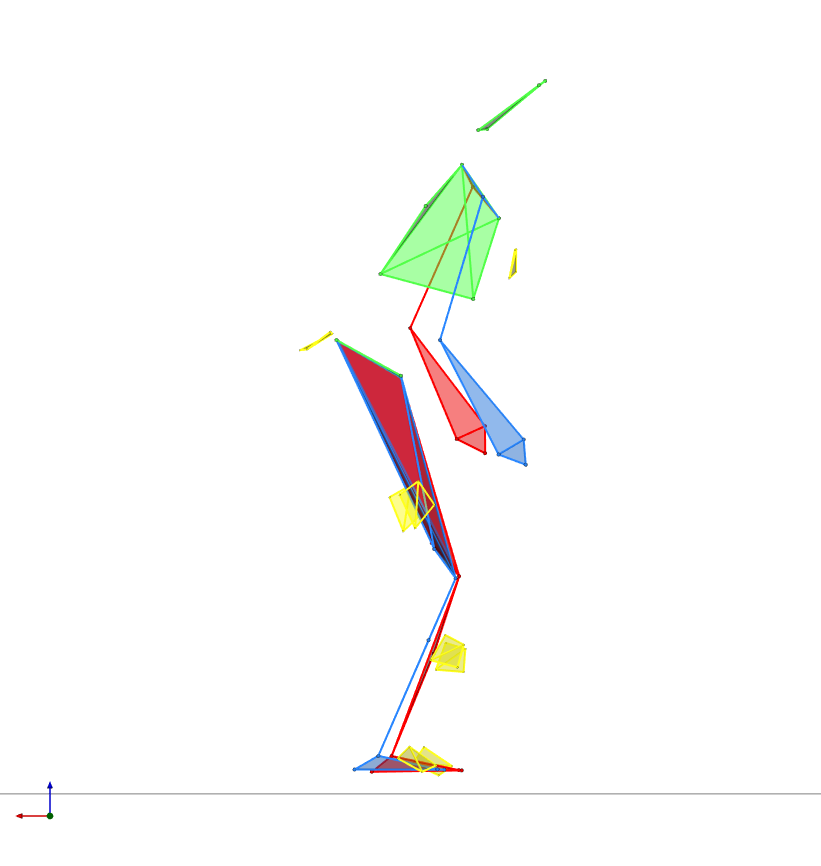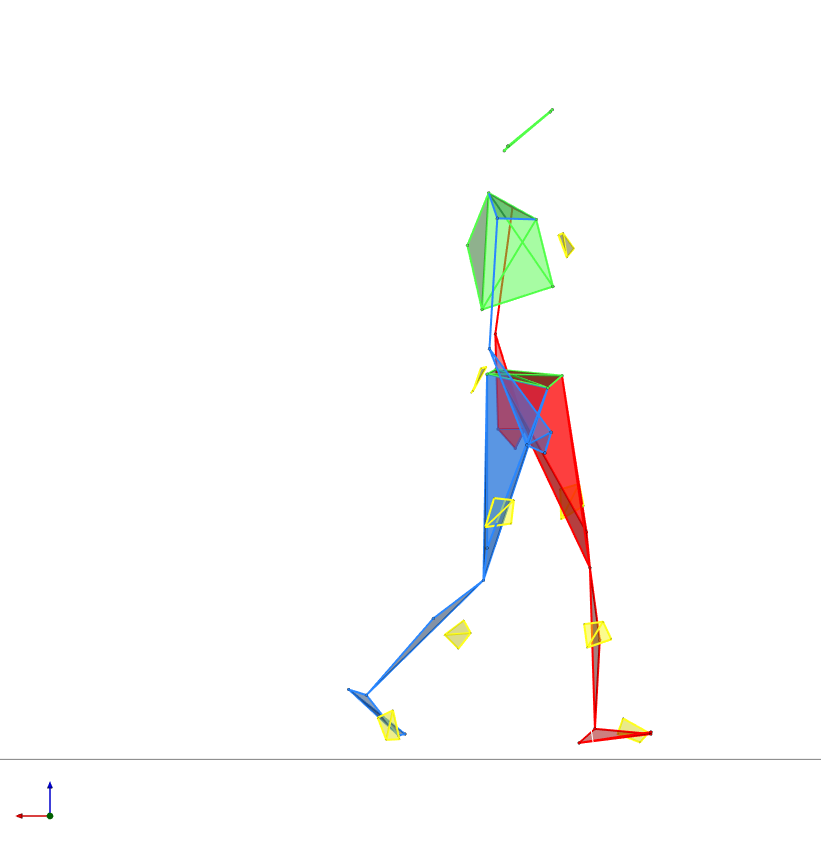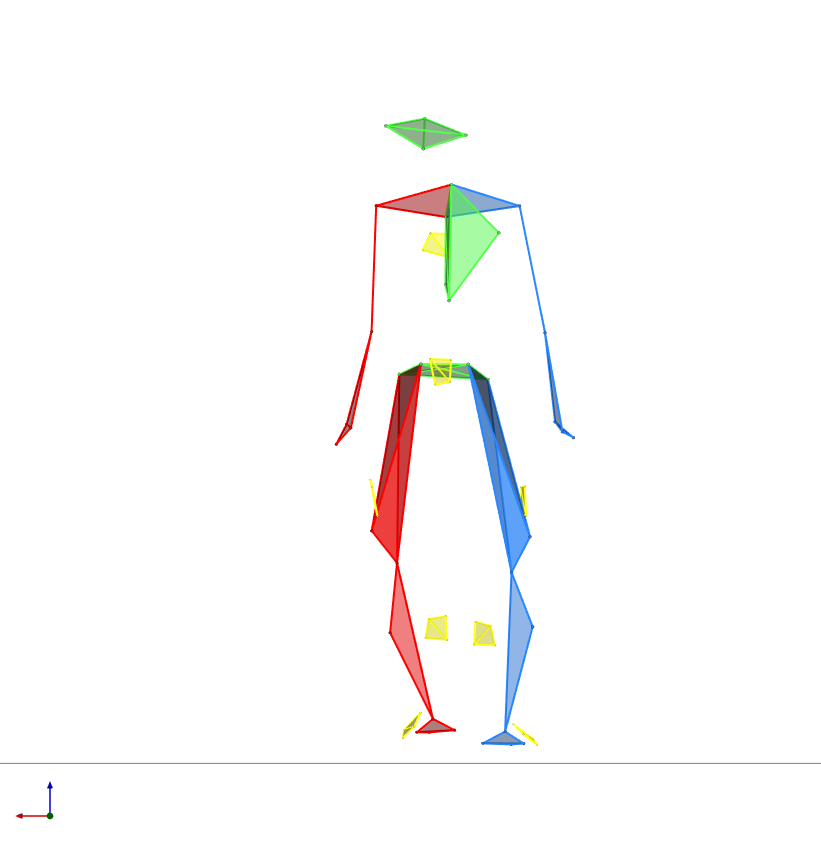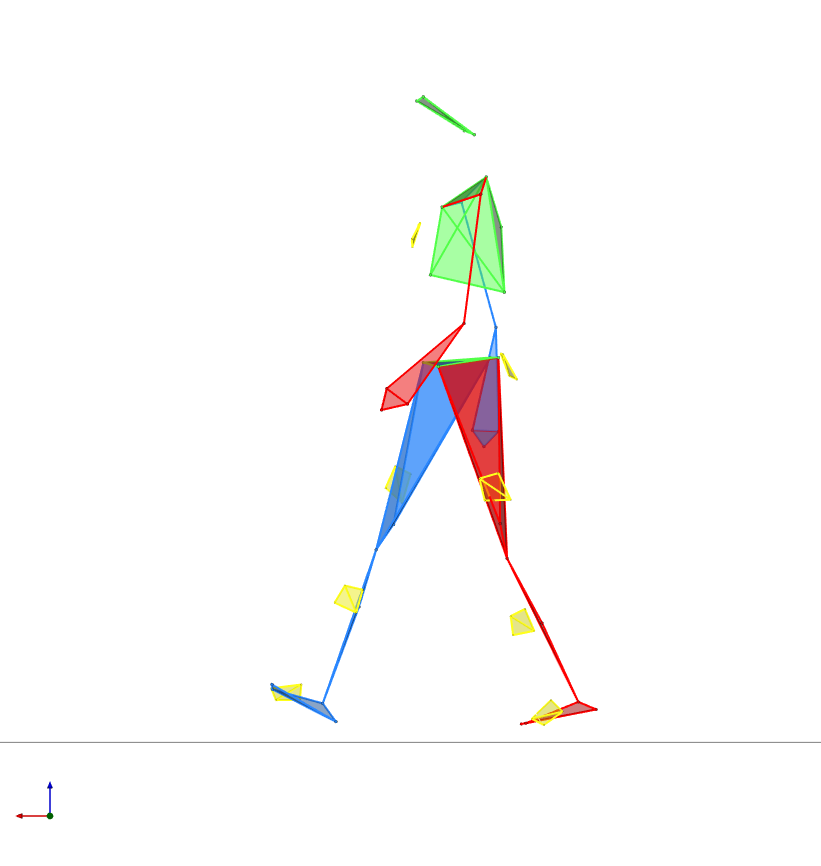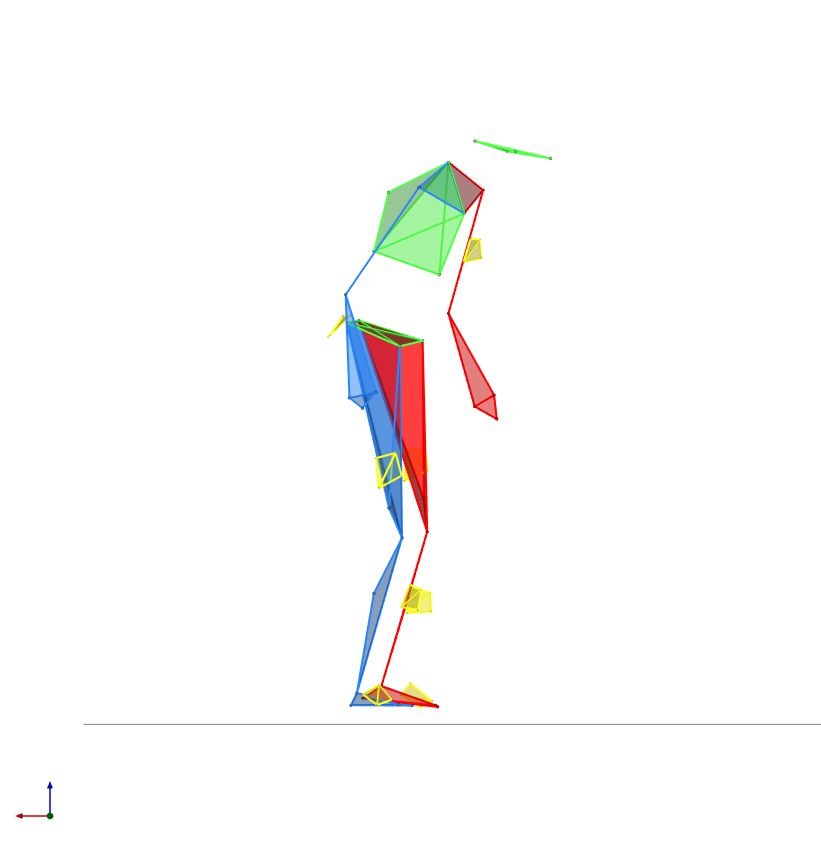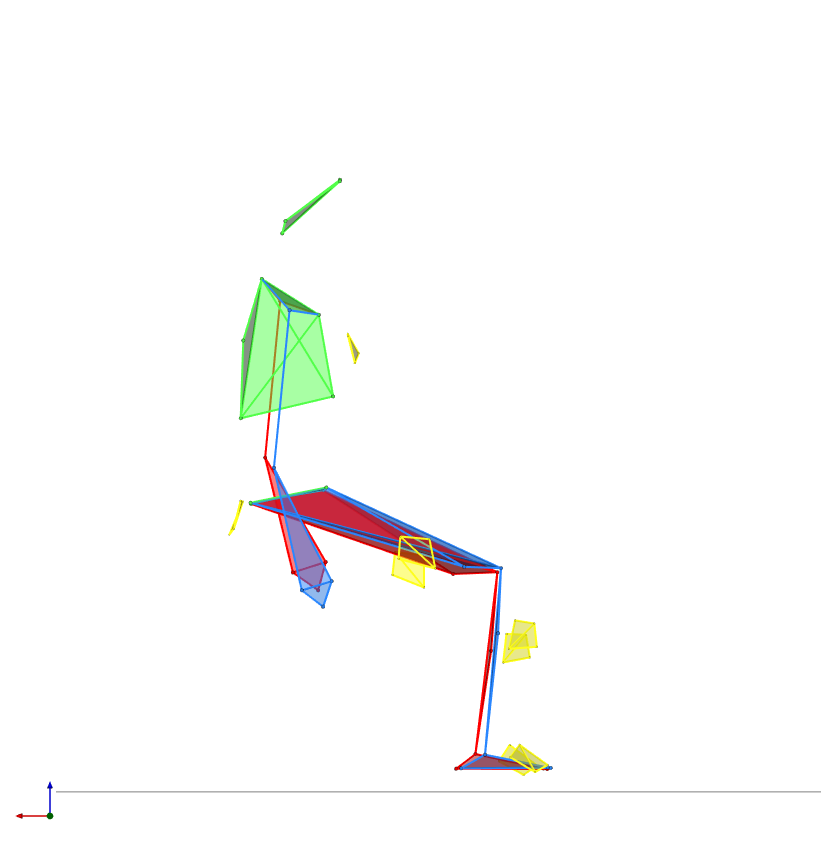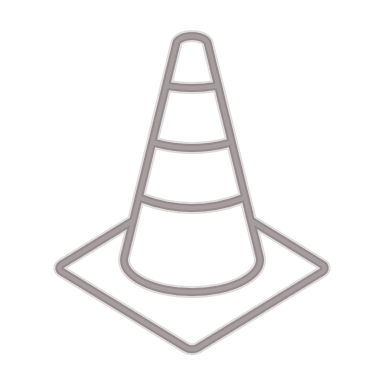 | - Sitting position : arms at the side of the body - No instruction to get up - No instruction to turn | *Stand up and walk 3 meters to the cone, turn around and walk back to the stool, turn to sit down and sit down* |
